# Is Systemic Immunosuppression a Risk Factor for Oral Cancer? A Systematic Review and Meta-Analysis

**DOI:** 10.3390/cancers15123077

**Published:** 2023-06-06

**Authors:** Romeo Patini, Massimo Cordaro, Denise Marchesini, Francesco Scilla, Gioele Gioco, Cosimo Rupe, Maria Antonietta D’Agostino, Carlo Lajolo

**Affiliations:** 1 Department of Head, Neck and Sense Organs, School of Dentistry, Catholic University of Sacred Heart, Fondazione Policlinico Universitario “A. Gemelli”—IRCCS Rome, 00135 Rome, Italy; romeo.patini@unicatt.it (R.P.); massimo.cordaro@unicatt.it (M.C.); denise.marchesini01@icatt.it (D.M.); francesco.scilla01@icatt.it (F.S.); cosimorupe@gmail.com (C.R.); carlo.lajolo@unicatt.it (C.L.); 2 Department of Geriatric and Orthopedic Sciences, Catholic University of Sacred Heart, Fondazione Policlinico Universitario “A. Gemelli”—IRCCS Rome, 00135 Rome, Italy; mariaantonietta.dagostino@unicatt.it

**Keywords:** immunosuppression, oral cancer, systematic review, meta-analysis

## Abstract

**Simple Summary:**

Immunosuppression is a medical condition in which a person’s immune system is unable to function properly, or it does not function at all. It is a well-known fact that an ill-functioning immune system can favor the generation and development of potentially malignant lesions, autoimmune and allergic diseases, and even neoplasms. At present, the amount of risk for the development of oral cancer in immunosuppressed patients has not been quantitatively reported. Such a topic has been investigated, revealing that immunosuppression increases the risk of developing cancer from 0.2% to 1% (95% CI: 0.2% to 1.4%), giving further importance to the accurate follow-up of this category of patients.

**Abstract:**

Even if the relationship between immunosuppression and increased incidence of systemic cancers is well known, there is less awareness about the risk of developing oral cancer in immunosuppressed patients. The aim of this review was to evaluate the association between immunosuppression and the development of oral cancer. Two authors independently and, in duplicate, conducted a systematic literature review of international journals and electronic databases (MEDLINE via OVID, Scopus, and Web of Science) from their inception to 28 April 2023. The assessment of risk of bias and overall quality of evidence was performed using the Newcastle–Ottawa Scale and GRADE system. A total of 2843 articles was identified, of which 44 met the inclusion criteria and were included in either the qualitative or quantitative analysis. The methodological quality of the included studies was generally high or moderate. The quantitative analysis of the studies revealed that immunosuppression should be considered a risk factor for the development of oral cancer, with a percentage of increased risk ranging from 0.2% to 1% (95% CI: 0.2% to 1.4%). In conclusion, the results suggest that a constant and accurate follow-up should be reserved for all immunosuppressed patients as a crucial strategy to intercept lesions that have an increased potential to evolve into oral cancer.

## 1. Introduction

According to official data from the World Health Organization (WHO, Geneva, Switzerland), 377,713 new cases of oral and lip cancer were diagnosed in 2020, making it the 16th most common cancer in the world. It still has a severe prognosis today, as approximately 50% of oral and lip cancer patients will die in the 5 years following diagnosis, while the remaining 50% have aesthetic and functional relics that make their quality of life rather low. Historically, the main risk factors for this neoplasm are being male, having a diet low in vitamins, having MPDs, past/present viral infections, radiation exposure, having genetic predispositions and immunodeficiencies, and engaging in luxuriant habits such as smoking and alcohol and betel consumption [1].

Oral cancer treatment is challenging and requires a multidisciplinary approach with a team of specialists, which includes head and neck surgeons, radiation oncologists, medical oncologists, and oral oncologists [2].

Although surgery is the most common initial definitive treatment for the majority of oral cancers, adjunctive radiotherapy (RT), with or without chemotherapy (CT) may be performed [3].

The immune system performs numerous functions, among which its primary functions are defense against infections, self-control and immunosurveillance at the onset and during the proliferation of solid and liquid cancers, identifying and suppressing genetically modified cells that have already passed the normal checkpoints, and the intracellular control of proliferation. The possible role of the immune system in the development of cancers has been defined in the theory of “immune surveillance”, which configures the active role of the immune system in preventing the onset of cancers [4].

Immune surveillance against cancer is the process in which the immune system identifies cancerous and/or precancerous cells and eliminates them.

According to the most recent findings, the immune system can play a role in preventing tumors, throughout different mechanisms. First, the virus-induced tumors can be prevented when a functioning immune system can eliminate or suppress viral infections. Second, this action against pathogens may cause a prompt resolution of inflammation, preventing the establishment of an inflammatory environment, which is a risk factor for carcinogenesis [5]. Third, the immune system can identify and eliminate tumor cells on the basis of their expression of tumor-specific antigens. Therefore, the theory of immunosurveillance is essentially based on two generally accepted claims: (I) most cancers are antigenic (an obvious requirement for immunological recognition) and (II) such antigenic differences can, “under appropriate conditions”, elicit an immune response [4].

Despite immune surveillance, cancers develop even in the presence of a functioning immune system, and therefore, currently, we speak of “cancer immunoediting”, a term which is used to describe the evolution of tumors, wherein tumor cells become less effectively recognized and killed by the immune system [6,7].

A first consideration concerns the definition that is used for patients with disorders of the immune system. The terms immunosuppression and immunodeficiency are often used interchangeably. This confusion is related to the subtle nuance that separates them. It could be specified that immunosuppression identifies a medical condition of a general malfunction of the immune system. Immunodeficiency, on the other hand, classifies the severity of this physical deficit according to two categories: primary and secondary.

Immunosuppression is a pathological condition characterized by the inhibition of one or more components of the immune system, whether natural or acquired, resulting in the impossibility of a person’s immune system to function properly. However, currently, there is no description illustrating the relationship between immunoediting and immunosuppression. The incorrect functioning of the immune system can favor the development of autoimmune and allergic diseases or neoplasms. Immunodeficiencies are divided into primary (if they are derived from congenital defects) and secondary (if they are derived from infections or pharmacological treatments) classifications. This condition involves the onset of infections that develop and recur very often, manifesting themselves in a more serious and longer-lasting form.

Among the many alterations of the immune system, immunodeficiency can be caused by numerous and different causes, and it can involve acquired or innate immunity, both in the humoral and cellular components, as follows: innate pathologies (e.g., agammaglobulinemia linked to the X sex chromosome, one common variable immunodeficiency, severe combined immunodeficiency, DiGeorge syndrome, and congenital hypogammaglobulinemia), systemic diseases (e.g., autoimmune diseases, diabetes, chronic infections, and solid and liquid malignancies, such as leukemia, lymphoma, and multiple myeloma), and pharmacological therapies (e.g., chemotherapy, antirheumatics, immunosuppressants, and glucocorticoids), which are the main causes of immunodeficiency [8,9].

By definition, immunodeficiency is characterized by a functional deficit of the immune system (either congenital or acquired). Immunosuppression is a pathological condition characterized by the inhibition of one or more components of the immune system (natural or acquired), and it occurs following an intercurrent disease or autoimmune pathologies [10]. Immunosuppression also refers to pharmacological treatment with immunosuppressive drugs capable of inhibiting an immune system response [11]. Therefore, immunocompromised patients have a reduced ability to fight infections and other diseases.

Numerous studies have shown that in immunosuppressed subjects, there is a higher incidence of cancers than in a population with normal immunity [12]. The increased susceptibility to infections (i.e., HPV, candida, Helicobacter pylori, etc.) and the reduced immune response to infections in immunosuppressed subjects could represent a further mechanism that favors the onset of neoplasms. Furthermore, immunosuppression is, at the same time, one of the risk factors for the onset of oncological pathologies, but it is also a condition that could favor the loco-regional and distant growth and spread of cancers. In fact, the literature shows that immunosuppression is not only a risk factor for the genesis of a cancer but also a factor for the prognosis of its course [13].

Although the relationship between immunosuppression and the increased incidence of systemic cancers is now well documented, currently, it is not clear how much the risk of developing oral cancer increases in immunosuppressed subjects and what effect immunosuppression has on prognosis in terms of survival. The purpose of this systematic review was, therefore, to evaluate the association and the possible correlation between the state of depression of the immune system and the development of oral cancer through the evaluation of the incidence of oral cancer in patients with systemic immunosuppression and to compare that to data from official databases (Globocan, WHO), which lacked precise data on non-immunosuppressed subjects.

## 2. Materials and Methods

In the present systematic review, the adopted protocol followed the Preferred Reporting Items for Systematic Reviews and Meta-Analyses (PRISMA) statement. The review protocol was registered in PROSPERO database (CRD42021243898).

### 2.1. PICOS Question

The following question was developed according to the population, intervention, comparison, outcome, and study design (PICOS).

Population: immunosuppressed patients who later developed oral cancer were included in this systematic review.

Intervention: patients with systemic immunodepression due to various factors (immunodepression, malnutrition, infections, autoimmune diseases, genetic immunosuppression, immunosuppression as a consequence of immunosuppressive therapy or radiotherapy, and oncologic immunosuppression) who subsequently developed oral cancer were considered.

Comparison: the rates of development of oral cancer in non-immunosuppressed patients and the rates of development of oral cancer in immunosuppressed patients were compared.

Outcome: the primary outcome was to evaluate the incidence of oral carcinoma in immunosuppressed patients.

Study design: cohorts, case controls, cross-sectional studies, and randomized clinical trials (RCTs) with no fewer than 10 patients were included. All case reports, case series with less than 10 patients, in vitro or in vivo studies based on animals, systematic reviews, letters to the editor, cases of oral cancer related to human papilloma virus (HPV), and articles published in languages other than Italian, English, and Spanish were excluded.

### 2.2. Focused Question

The question on which attention was focused was formulated on the basis of the PICOS criteria: “Do immunosuppressed patients have a higher rate of development of oral cancer than healthy patients?”.

### 2.3. Research

The research was conducted on three databases (MEDLINE via OVID, Scopus, and Web of Science) from the start of their activity in May 2022, using a combination of key words and MeSH terms as follows: ((immunosuppression OR malnutrition OR infections OR autoimmune disease OR X-linked agammaglobulinemia OR common variable immunodeficiency OR selective immunoglobulin A deficiency OR hyper IgM syndrome OR DiGeorge syndrome OR severe combined immunodeficiency OR Wiskott–Aldrich syndrome OR acquired immunodeficiency syndrome OR AIDS OR immunosuppressive therapy OR radiotherapy OR “other systemic cancers” OR leukaemia OR lymphoma) AND “Oral Cancer”), (“Oral Carcinoma” AND (immunosuppression OR malnutrition OR infections OR autoimmune disease OR X-linked agammaglobulinemia OR common variable immunodeficiency OR selective immunoglobulin A deficiency OR hyper IgM syndrome OR DiGeorge syndrome OR severe combined immunodeficiency OR Wiskott–Aldrich syndrome OR acquired immunodeficiency syndrome OR AIDS OR immunosuppressive therapy OR radiotherapy OR “other systemic cancers” OR leukaemia OR lymphoma)), and (“Oral Neoplasms” AND (immunosuppression OR malnutrition OR infections OR autoimmune disease OR X-linked agammaglobulinemia OR common variable immunodeficiency OR selective immunoglobulin A deficiency OR hyper IgM syndrome OR DiGeorge syndrome OR severe combined immunodeficiency OR Wiskott–Aldrich syndrome OR acquired immunodeficiency syndrome OR AIDS OR immunosuppressive therapy OR radiotherapy OR “other systemic cancers” OR leukaemia OR lymphoma)). The date of the last search was 28 April 2023.

### 2.4. Manual Search

A manual search of articles published between 2002 and 2022 in the following peer-reviewed journals was performed: *Oral Oncology*, *Oral Diseases*, *Lancet Oncology,* and *Journal of Hematology and Oncology*.

### 2.5. Search of Unpublished Articles

Unpublished literature was searched in the U.S. National Institutes of Health clinical trials registry and the European Multidisciplinary Database to identify incumbent studies and grey literature. In addition, bibliographic references of all included articles and reviews were similarly checked to identify additional potentially relevant studies and increase the sensitivity of the search.

### 2.6. Study Selection

Based on the inclusion criteria, two authors independently and in duplicate (D.M. and F.S.) analyzed the titles and abstracts of the articles found. The authors retrieved the full versions of articles whose titles and abstracts appeared to meet the inclusion criteria or those, which reported insufficient data to make a clear decision. Next, the two authors independently read the full texts to determine whether the articles met these criteria. In cases where the two authors disagreed, agreement was sought through a comparison between the two, and when a solution could not be reached, a third senior author (R.P.) stepped in.

To calculate the agreement between the reviewers, Cohen’s kappa coefficient was used. The level of agreement was considered excellent when k was greater than 0.75, fair to good when it was between 0.40 and 0.74, and poor when it was less than 0.4 [14].

All articles that met the inclusion criteria were subjected to data extraction and quality assessments. All irrelevant articles were excluded, and the reasons for exclusion were as described.

### 2.7. Extraction Data

The data were collected using a purpose-built data extraction form. In cases where the publication did not provide all the necessary data, the corresponding author was contacted by e-mail to obtain the missing data. In the event that the two authors disagreed about one of the publications, a discussion was opened, which, in cases of disagreement, required the intervention of the third author.

In cases of redundant publications, the most recent article and the one with the largest follow-up were included.

### 2.8. Quality Assessment

The risk of bias in the included studies was independently assessed in duplicate by two authors as part of the data extraction process.

An assessment of risk of bias was undertaken using the Newcastle–Ottawa Scale (NOS) [15]. The presence of each parameter was recorded with a green mark, while absence was recorded with a red mark (0). Papers with 1–3 green marks were classified as high risk of bias, those with 4–6 green marks were classified as medium risk, and those with 7–9 green marks were classified as low risk. A supplemental analysis was performed independently by the two examiners regarding the overall quality of the evidence for any performed meta-analysis using the Grading of Recommendations, Assessment, Development, and Evaluations (GRADE) system [16]. Any disagreement between the two reviewers (D.M. and F.S.) was solved by discussion with the author supervisor (R.P.).

Publication bias was assessed through a funnel plot, which was made using Excel software (Microsoft Excel^®^).

### 2.9. Heterogeneity Assessment

The OpenMeta software was used for assessing the heterogeneity of the studies included in any conducted meta-analysis (OpenMeta, Inc.©, Zug, Zug, Switzerland). The authors calculated the comparability of the observed proportions across the results with chance alone using the I2 test. In cases where the *p*-value was <0.1, the heterogeneity was considered significant. Moreover, the same test was considered as a measure of heterogeneity across studies, following the subsequent scheme [17]: 0–40%, negligible; 30–60%, moderate; 50–90%, substantial; and 75–100%, considerable.

### 2.10. Data Analysis

Descriptive characteristics of the studies are expressed as means/medians and/or frequencies, as appropriate, depending on the variables.

Meta-analyses were performed only when there were studies comparing similar groups and reporting the same outcomes. In such cases, the meta-analyses were performed with a fixed-effect model. A random-effect model was used only in the case of not-negligible heterogeneity across the included studies (>50%).

A forest plot was created to illustrate the effects on the meta-analysis of individual studies and the overall estimate. OpenMeta-analyst [18] was used to perform all analyses. The cut-off value of significance was set at *p* < 0.05.

## 3. Results

### 3.1. Study Selection

**Figure 1:**
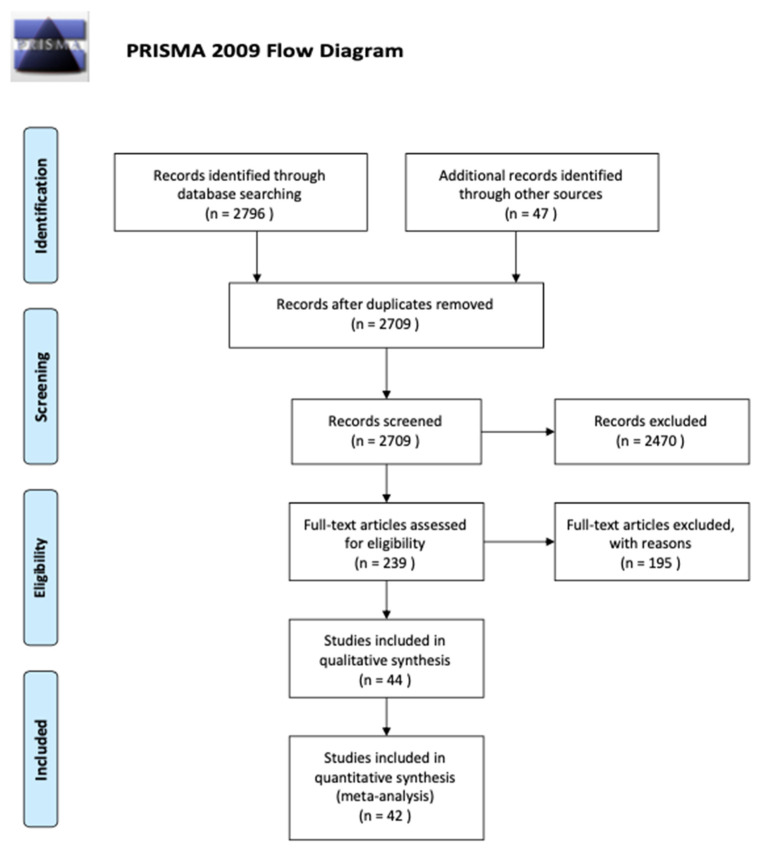
Flowchart of the selection of the studies for the review.

A flowchart of the search strategy and study selection is shown in Figure 1.

A total of 2843 articles was identified, with 2796 found through electronic searches and 47 found through other sources. Out of the 2709 studies that resulted after removal of the duplicates, 2470 were excluded as a result of title and abstract reading (inter-reader agreement, k = 0.78). Eventually, out of the 239 articles that remained to be evaluated in the full-text, 44 met the inclusion criteria and were included in either the qualitative or quantitative analyses (meta-analysis); in contrast, 195 were excluded. All information about full-text articles excluded, with reasons are included in the Supplementary Materials File (Table S1).

### 3.2. Study Characteristics

The characteristics of the included studies are summarized in Table 1.

Both prospective (five studies) and retrospective (nine studies) cohort studies were included in the review. Twenty-four studies presented data from national registries, and therefore, they were analyzed separately. In addition, six studies presented results related to a single immunosuppression condition, namely, graft-versus-host disease, and for this reason, they were analyzed separately, as this condition is, itself, a potentially malignant disorder of the oral cavity. All studies were conducted in an institutional environment.

### 3.3. Assessment of the Risk of Bias

**Figure 2:**
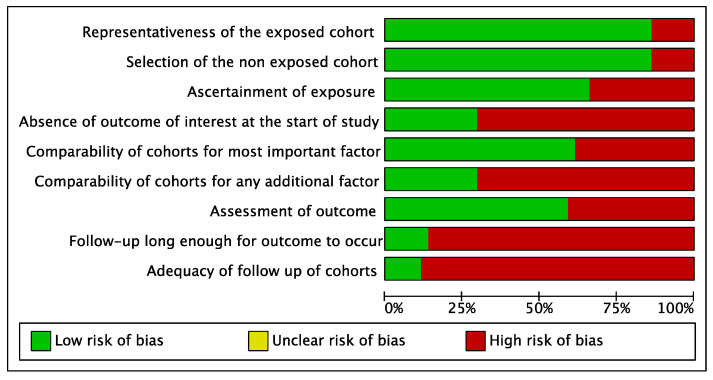
Risk of bias graph.

**Figure 3:**
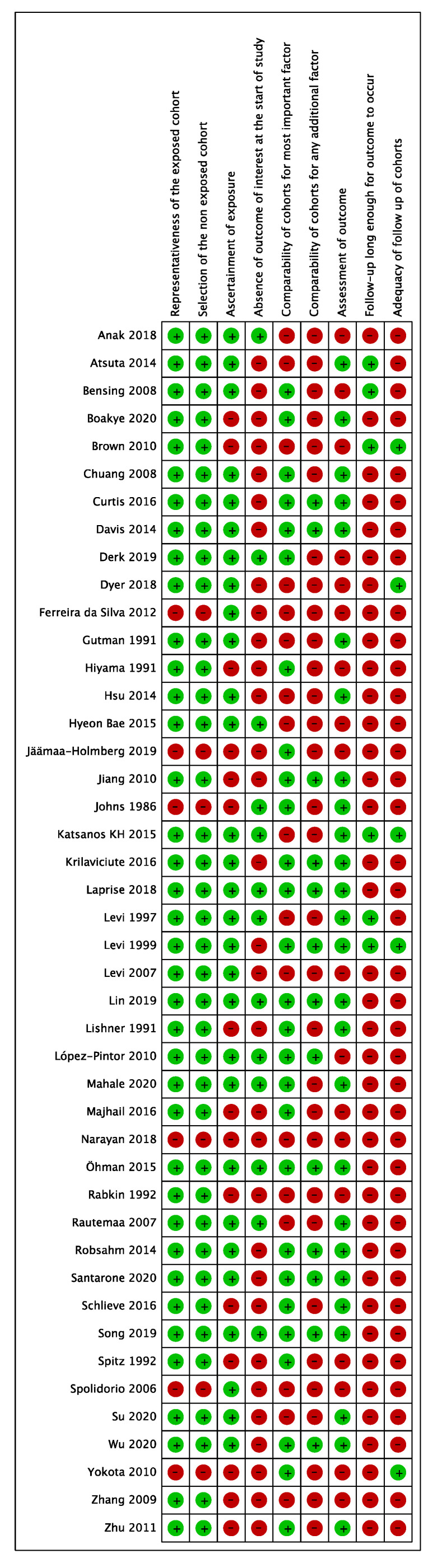
Risk of bias summary [19,20,21,22,23,24,25,26,27,28,29,30,31,32,33,34,35,36,37,38,39,40,41,42,43,44,45,46,47,48,49,50,51,52,53,54,55,56,57,58,59,60,61,62].

The risk of bias is summarized in Figure 2 and Figure 3. The methodological quality of the included studies was high for 12 studies [19,22,24,25,28,31,32,33,36,51,53,58], moderate for 26 studies [20,21,23,25,26,29,30,32,34,37,38,39,40,41,42,43,44,45,46,47,48,49,50,52,54,55,56,57,60,61,62], and low for six studies [23,26,27,35,48,59].

**Figure 4:**
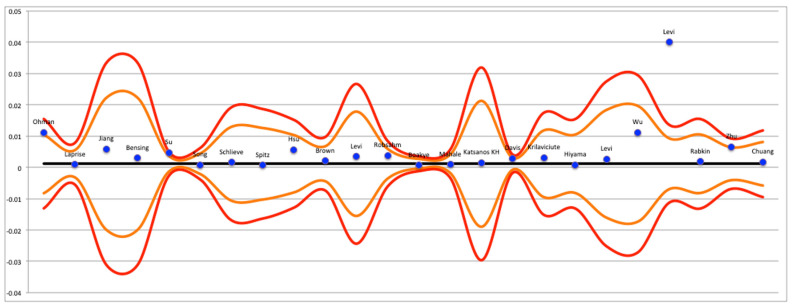
Funnel plot of studies with data from national registries [20,23,26,31,32,33,34,35,36,37,38,39,40,41,42,44,45,46,47,48,49,50,57,59].

**Figure 5:**
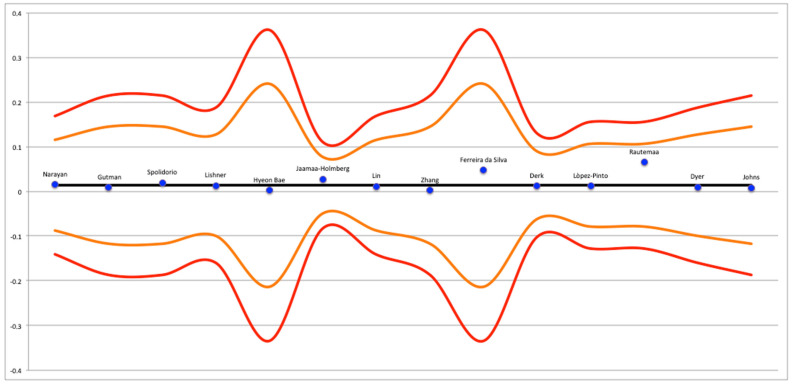
Funnel plot of studies with data not from national registries [19,21,22,24,25,27,28,29,30,43,54,58,60].

**Figure 6:**
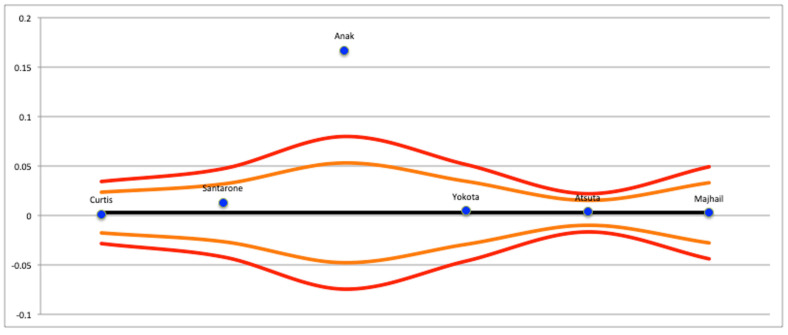
Funnel plot of studies with data about GVHD [51,52,53,55,56,61].

The results regarding publication bias are presented in Figure 4, Figure 5 and Figure 6. Significant publication bias was found in the studies that presented results related to Graft Versus Host Disease (GVHD) and the national registries. The Grading of Recommendations, Assessment, Development, and Evaluations (GRADE) system provided information on the certainty of the conclusions and the strength of the evidence (Table 2). Although the meta-analyses drew conclusions from cohort studies, which are considered to be among the best-available evidence, they were considered to have only moderate strength of evidence because of the presence of at least one study with a high risk of bias and very wide confidence intervals.

### 3.4. Results of the Meta-Analyses

**Figure 7:**
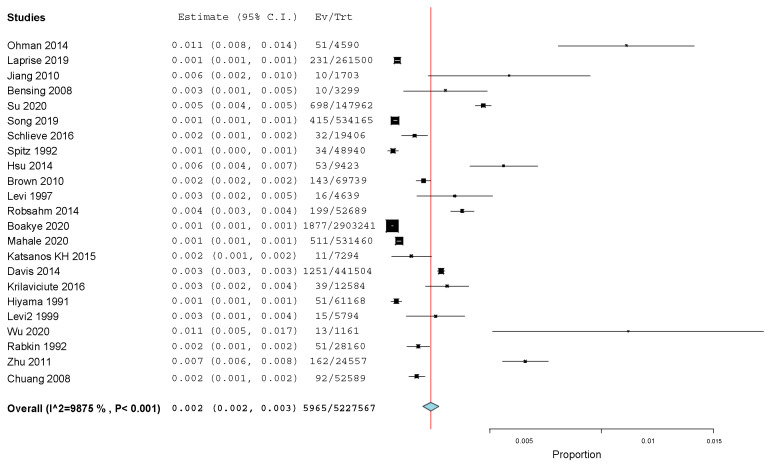
Meta-analysis related to data coming from national registries [20,23,26,31,32,33,34,35,37,38,39,40,41,42,44,45,46,47,48,49,50,57,59].

As reported earlier, three separate meta-analyses were conducted. The meta-analysis related to the national registries (Figure 7) was conducted on 23 studies with a total of 5,227,567 patients and found an “untransformed proportion” (PR) of 0.2% (95% CI: 0.002–0.003) (*p*-value of <0.001).

**Figure 8:**
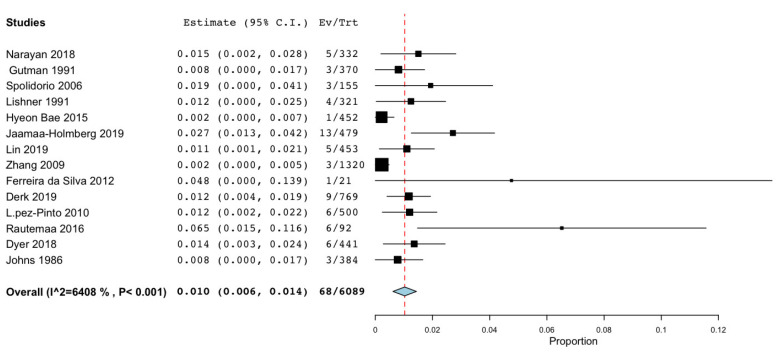
Meta-analysis related to data not coming from national registries [19,21,22,24,25,27,28,29,30,43,54,58,60,62].

The meta-analysis concerning data not derived from the national registries (Figure 8) was conducted on 15 studies with a total of 6997 patients and found an “untransformed proportion” (PR) of 1% (95% CI: 0.006–0.014) (*p*-value of <0.001).

**Figure 9:**
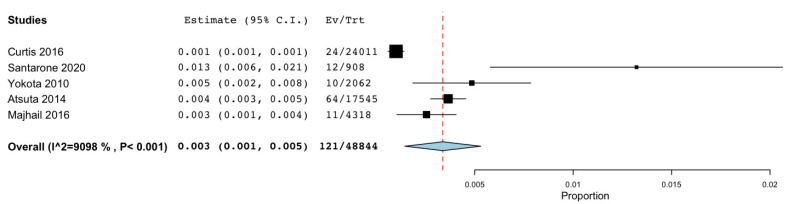
Meta-analysis regarding data about GVHD [51,52,53,56,61].

The meta-analysis regarding data about GVHD (Figure 9) was conducted on six studies with a total of 49,285 patients and found an “untransformed proportion” (PR) of 0.3% (95% CI: 0.001–0.005) (*p*-value of < 0.001).

The meta-analyses conducted on the three groups of patients revealed a general increased risk of developing an oral cancer in immunosuppressed populations. Such risk ranges from 0.2% to 1% depending on whether data from national registries are considered. In immunosuppressed patients, this evidence emphasizes the need to provide for a careful follow-up of suspicious lesions and potentially malignant disorders of the oral cavity.

## 4. Discussion

### 4.1. Summary of the Main Findings

The close relationship between the immune system and cancer immunoediting has been documented for many years for numerous cancers, including oral carcinoma, and this systematic review highlighted an incidence of oral carcinoma in immunosuppressed patients of 200 new cases per 100,000. If this is compared to data from registries on the incidence of oral cancer in the general population, which is approximately 4.1 per 100,000 subjects (ASR incidence = 4.1 per 100,000), immunosuppressed subjects have a risk of developing oral cancer that is 50 times higher than the general population. These raw data emphasize the need to establish clinical protocols for primary prevention and screening in all immunosuppressed subjects, likely with tailor-made protocols that depend on the cause of immunosuppression and the severity of the immunosuppression.

Some considerations of a methodological nature that emerged from this systematic review should be made in light of the literature. A first consideration concerns the definition that is used for patients with disorders of the immune system. The terms immunosuppression and immunodeficiency are often used interchangeably. This confusion is related to the subtle nuance that separates them. It could be specified that immunosuppression identifies a medical condition involving a general malfunction of the immune system, whereas immunodeficiency classifies the severity of this deficit into primary and secondary in relation to the cause. Furthermore, an aspect still unresolved concerns the identification of clinical and/or instrumental parameters that can identify the state of immunosuppression (considering both innate and acquired immunity, both cellular and humoral) and classify it in relation to the severity of the immunosuppression.

The present systematic review demonstrated that immunosuppression should be considered a risk factor for the development of oral cancer, with a percentage of increased risk ranging from 0.2% to 1% (95% CI: 0.2% to 1.4%). Considering the main causes of immunosuppression reported in the selected articles, there are some interesting considerations. In fact, in this systematic review, the authors decided to divide the results from the included papers into three main groups: the results derived from the literature analysis of the main reasons for immunosuppression (not from national registries), those from articles related to GVHD, and those from the registry analysis, which depict an increased risk of 1% (95% CI: 0.6% to 1.4%), of 0.3% (95% CI: 0.1% to 0.5%), and of 0.2% (95% CI: 0.2% to 0.3%), respectively. Articles referring to states of malnutrition were not included in this review, as they did not report adequate information regarding immune status.

### 4.2. Organ Transplantation

Organ transplantation, in particular, kidney transplantation, represents one of the main causes of immunosuppression most frequently associated with the onset of oral cavity neoplasms. The increased life expectancy of transplant recipients exposes them to prolonged immunosuppressive therapy (mainly cyclosporine), which is necessary to avoid the phenomenon of transplant rejection. In the study conducted by López-Pintor [62], 500 kidney transplant patients were recruited, and during follow-up, six cases of oral cancer were reported out of 500 patients (incidence of 1 patient per 100 subjects).

The same trend was seen for patients undergoing heart transplantation (HTx). Due to new techniques introduced in transplant surgery, survival after heart transplantation has improved significantly in recent decades. In the study conducted by Jääma-Holmberg (2019) [25], the risk of oral cancer after organ transplantation was two to four times higher than that of the general population, becoming one of the main long-term complications in this group of patients. Furthermore, it would appear that oral cancer occurs with a higher frequency in subjects who have undergone thoracic organ transplantation rather than those who have undergone abdominal organ transplants (i.e., liver and kidney). This different risk of oral cancer in relation to the type of organ transplanted could be partly related to the different pharmacological regimens adopted and partly linked to the underlying pathologies that lead to the need for transplants. Further studies should stratify the risk of oral cancer in relation to the type of organ transplanted.

### 4.3. Other Cancers

Another cause of immunosuppression associated with a greater risk of developing oral cavity cancer is represented by the treatment of thyroid neoplasms. The number of newly diagnosed cases of thyroid cancer has increased in recent years due to technological advances and the spread of cytological tests for early diagnosis. Patients who underwent partial or total thyroidectomy and those who received radio-iodine treatment for the treatment of thyroid cancer reported an increased risk of developing oral cancer. The study by Hsu et al. (2014) [40] showed an increased association between thyroid cancer and subsequent head and neck cancer. This association found that its biochemical-molecular explanation was related to the intrinsic carcinogenic action of radio-iodine, which can possibly be enhanced by pre-existing molecular genetic mutations in a framework of immunological impairment linked to the partial or total removal of the thyroid.

### 4.4. Infectious Agents

Other known causes of immunosuppressive states are infectious agents (i.e., HCV, HIV, and HPV). This literature review reported only one study, which was conducted by Su et al. [49] that highlighted an incidence of 698 cases of carcinoma out of 147,962 patients. The risk of oral cancer appears to be lower in HCV patients receiving pegylated interferon (PEG-IFN) therapy than that of untreated HCV patients. Further studies should investigate the role of HCV infection in oral cancer oncogenesis, with particular attention paid to the type of therapy administered to patients.

Studies investigating the role of HIV as a cause of immunosuppression were not included in this review. In fact, it is known that HIV infection causes a depletion of CD4+ T lymphocytes, with consequent impairment of the immune system. Acquired immunodeficiency could, therefore, lead to an increased risk of oral cancer. The study conducted by Precious K. Motlokwa et al. (2022) on an oral cancer population in sub-Saharan Africa did not show an increased risk of carcinogenicity in a group of HIV-infected patients [63]. This could be partly explained by new antiretroviral therapies, which allow clinicians to gain control of HIV infections and, therefore, reduce the impairment of patients’ immune systems. Further studies are needed to evaluate whether there is a real risk in HIV-positive patients and whether there are associated risk factors (CD4 T lymphocyte count or traditional antiretroviral therapies vs HAART).

### 4.5. Hematopoietic Stem Cell Transplantation (HSC)

Within the selected articles, it was possible to identify a group of articles conducted on patients undergoing hematopoietic stem cell transplantation (HSC), which now represents an essential therapy for the treatment of various haemato-lymphoproliferative diseases and other benign conditions (multiple myeloma, lymphomas, autoimmune disorders, etc.). In the study conducted by Santarone et al. (2020) [56], patients undergoing HSC transplantation reported the incidence of developing a malignancy at double the rate of the general population. In support of this, Dyer and colleagues [54] also found a similar incidence rate in patients undergoing HSC transplantation, underlining the importance of regular follow-ups with patients.

Furthermore, GVHD is among the adverse events associated with HSC transplantation. This clinical condition represents an adverse immunological phenomenon following HSC transplantation. GVHD oral lesions are among the so-called potentially malignant disorders, as they have a greater risk of neoplastic degeneration than healthy mucosa. Furthermore, the most frequently used therapy in the treatment of GVHD involves the use of immunosuppressive agents (e.g., both topical and high potency systemic corticosteroids and calcineurin inhibitors), which, although they reduce the inflammatory component of GVHD lesions, could increase the risk of developing a secondary malignancy. The risk of developing malignancy in patients with chronic GVHD was significantly increased compared with the general population, with a standard incidence ratio (SIR) of 1.8 and a 95% confidence interval (95% CI) of 1.5–2.0. The risk is much higher for cancer of the oral cavity (SIR = 15.7, 95% CI, 12.1–20.1), cancer of the esophagus (SIR = 8.5, 95% CI, 6.1–11.5), colon cancer (SIR = 1.9, 95% CI, 1.2–2.7), skin cancer (SIR = 7.2, 95% CI, 3.9–12.4), and cancers of the nervous system (SIR = 4.1, 95% CI, 1.2–8.4). The risk of developing oral, esophageal, or skin cancer appears to have a maximum incidence 1 year after transplantation [61].

### 4.6. Strengths and Limitations of the Present Systematic Review

Finally, the data obtained from this systematic review were partly extrapolated from the analysis of national registers from China, Japan, Republic of Korea, India, Taiwan, and Nordic Scandinavian countries. As these databases have a large amount of data, they can lead to significant statistical variations capable of creating very significant discrepancies in the results. In light of this, a meta-analysis dedicated solely to the analysis of the data obtained from these registries was conducted in this systematic review. It is also known that cancer of the oral cavity has a notably high incidence in the aforementioned countries (e.g., China and India) due to the different cultural and social habits. The funnel plot shown in Figure 4 revealed the presence of some studies with particularly discrepant data with respect to the confidence interval of the meta-analysis. Specifically, the study conducted by Levi et al. was discrepant to the funnel plot, and for this reason, it was removed from the statistical analysis and presented only in a qualitative form.

From a methodological point of view, all the studies included in this review had the main objective of investigating the incidence of cancer in other sites. Therefore, further prospective observational studies evaluating the occurrence of oral cancers in immunosuppressed patients as the main outcome while also taking into account the main risk factors of oral cancer that may influence this association (e.g., smoking, candida, HPV, and alcohol) are required. Moreover, it is essential to consider adequate follow-ups to avoid an underestimation of the real incidence of oral carcinomas. The time factor certainly plays an important role in the carcinogenic process, considering that a prolonged state of immunosuppression can increase the risk of the onset of neoplasms.

## 5. Conclusions

The results obtained from the systematic review indicated that immunosuppression is to be considered a risk factor for the development of oral cancer.

Particular attention and accurate follow-ups with all immunosuppressed patients are, therefore, essential in order to intercept clinical situations at an early stage that could evolve into oral cancer.

Further studies are needed to investigate the effective role of immunosuppression in carcinogenesis and to identify any risk factors.

## Figures and Tables

**Table 1:** Characteristics of the included studies.

Authors-Year	Study Setting	Study Design	No. Patients (Gender)	Cause of Immunodepression	No. Patients Who Delevoped Oral Cancer	% of Oral Cancer (Cancer/Tot)	Age (Mean)	Gender	Aim	Oral Cancer Site	Follow Up (Years)
				Organ Transplant							
Spolidorio, 2006 [19]	São Paulo Hospital	P	155 (120 M, 35 F)	Cyclosporin A or tacrolimus	3	1.93%	Unknown	NR	To determine the oral status of renal transplant recipients receiving cyclosporin A or tacrolimus as immunosuppressant	Lip	unknown
Jiang, 2010 [20]	Canadian Organ Replacement Register	R	1703 (1405 M, 298 F)	Heart transplantation	10	0.58%	54.4	NR	To assess the long-term risk of developing cancer among heart transplant recipients compared to the Canadian general population	NR	6.08 years
Lòpez-Pintor, 2010 [21]	Hospital Universitario 12 de Octubre, Madrid, Spain	R	500 (193 F, 307 M)	Renal transplantation	6	1.2%	57.33	M	To establish the incidence of lip cancer (LC) in a population of renal transplant patients (RTPs)	lip	18
Ferreira da Silva, 2012 [22]	Department of the federal university of Sergipe, Brazil	R	21 (7 F, 14 M)	Kidney transplantation	1	4.76%	42	M	To investigate oral lesions in kidney transplant patients	lip	2.5 (mean)
Ohman, 2014 [23]	Sahlgrenska University Hospital Register	R	4590 (2839 M, 1751 F)	Transplantation	51	1.11%	62	NR	To verify an increased risk of oral and lip cancer in solid organ transplantation patients	4 tongue, 5 salivary glands, 3 floor of mouth, 3 gingiva, palate, bucca, 34 lip	Median 6.3 years
Narayan, 2018 [24]	Medwin Hospitals, Telangana, India	P	332	Renal transplantation	5	1.50%	NR	NR	To identify the number of patients with renal transplant who developed second cancer	tongue	26
Jaamaa-Holmberg, 2019 [25]	NA	R	479 (381 M, 98 F)	Heart transplantation	13	2.71%	Unknown	NR	To demonstrate that cancer incidence in Finnish HTx-recipients is six times higher than in general Finnish population	7 lip, 4 tongue, 1 salivary glands, 1 non specified	Median 7.8 years
Laprise, 2019 [26]	The scientific Registry of transplant recipients	R	261,500 (174,475 M, 109,357 F)	Transplantation	231	0.09%	50	NR	To evaluate the incidence of lip cancer after solid organ transplantation	231 lip	Median 3.96 years
Lin, 2019 [27]	Changhua Christian Hospital	R	455-2 (453)	Liver transplantation	5	1.10%	56	1 F, 4 M	To identify the number of head and neck cancer in liver transplant recipients	3 tongue, 1 retromolar trigone, 1 buccal mucosa, 1 parotid gland	NR
				**Other** **Cancers**							
Johns, 1986 [28]	Johns Hopkins Medical Istitutions, Baltimore	R	384 (206 F, 178 M)	Salivary gland or thyroid gland malignancies	3	0.78%	NR	1 F, 2 M	To determine the exact risk of multiple primary neoplasms in patients with salivary gland or thyroid gland malignancies	3 salivary glands	10
Gutman, 1991 [29]	Tel Aviv Medical Center	P	370 (133 M, 237 F)	Melanoma	3	0.81%	60.5	F	To identify the number of patients with GVHD who developed second cancer	NR	Different based on stages
Lishner, 1991 [30]	Princess Margaret Hospital, Toronto	R	321	Non-Hodgkin’s lymphoma	4	1.24%	48	3 M, 1 unknown	To evaluate the incidence of second malignant tumors in patients with Non-Hodgkin’s lymphoma	3 tongue, 1 gingiva	At least 6 months
Hiyama, 1991 [31]	Department of field research, Osaka	R	61,168 (22,391 F, 38,777 M)	Stomach cancer	51	0.08%	NR	NR	To determine the risk of second primary cancer after diagnosis of stomach cancer in Osaka	NR	30
Spitz, 1992 [32]	National Cancer Institute	R	48,940 (F)	Cervix cancer	34	0.07%	NR	F	To evaluate the association between malignancies of the upper aerodigestive tract and uterine cervix	NR	11 years
Rabkin, 1992 [33]	National cancer institute, Belthesda	R	28,160 (25,295 F, 2865 M)	Anal and cervical carcinoma	51	0.18%	NR	NR	To determine the risk of second primary cancer following anal and cervical carcinoma	NR	NR
Levi, 1997 [34]	The Cancer Registries, Switzerland	R	4639	Skin Cancer	16	0.34%	74	NR	To evaluate the incidence of second primary cancers in patients with skin cancer	5 lip, 3 salivary gland, 8 mouth	23 years
Levi, 1999 [35]	University of Milan, Italy	R	5794	Lung carcinoma	15	0.26%	NR	NR	To determine the risk of second primary cancer in patients with lung carcinoma	NR	22
Levi, 2007 [36]	Universitè de Lausanne	R	1672 (424 F, 1248 M)	Esophageal cancer	67	4.00%	55	NR	To determine the risk of second neoplasms after esophageal cancer	NR	30
Chuang, 2008 [37]	Lyon, France	R	52,589 (19,110 F, 33,479 M)	Esophageal cancer	92	0.18%	NR	NR	To assess the risk of second primary cancers following a first primary esophageal cancer	NR	10
Brown, 2010 [38]	The National Cancer Institute’s Survival	R	69,739 (F)	Endometrial cancer	143	0.20%	62	F	To examine the risk of subsequent primary malignancies (SPMs) in women diagnosed with endometrial cancer.	NR	11.2 years
Zhu, 2011 [39]	Academy of Medical Sciences, Gansu, China	R	24,557 (6253 F, 18,304 M)	Treatment of esophageal cancer	162	0.66%	NR	NR	To determine the risk of second primary cancer after treatment for esophageal cancer	NR	34
Hsu, 2014 [40]	Taiwan’s National Health Insurance	R	9423 (1940 M, 7483 F)	Thyroid cancer	53	0.56%	NR	NR	To determine the association of thyroid cancer with other malignancies in Taiwan.	40 mouth, 13 salivary glands	NR
Robsahm, 2014 [41]	Cancer Registry of Norway	R	52,689 (28,069 CMM, 24,620 SCC)	Squamous cell carcinoma and melanomas	47 (CMM), 152 (SCC)	0.37%	NR	33 M, 14 F (CMM)/114 M, 38 F (SCC)	To examine the risk of a new primary cancer following an initial skin cancer	NR	10.1
Davis, 2014 [42]	University of Michigan Medical school	R	441,504 (M)	Prostate cancer	1251	0.28%	NR	NR	To determine the risk of second primary tumors in men with prostate cancer	NR	10
Hyeon Bae, 2015 [43]	Chonnam National University Hospital, Hwasun, Korea	R	452 (208 M, 244 F)	Melanoma	1	0.22%	Unknown	NR	To assess the presence of other primary cancer in patients with acral and non-acral melanomas	NR	No
Krilaviciute, 2016 [44]	National cancer institute, Vilnius, Lithuania	R	12,584 (8074 F, 4510 M)	Basal cell carcinoma	39	0.31%	NR	NR	To determine the risk of second primary cancer in basal cell carcinoma patients in Lithuania	14 lip, 25 other in oral cavity	14
Schlieve, 2016 [45]	University of Tennessee	R	19,406/849	Primary Non-head-neck cancer	32	80%/	67	NR	To determine the rate of second primary head and neck cancer development among patients with a primary cancer diagnosed outside of the head and neck region, to present the clinical characteristics of this population, and to determine if any variables are associated with survival.	11 gingiva, 7 tongue, 4 base of tongue, 4 buccal, 3 floor of mouth, 2 palate, 1 parotid	10 years
Boakye, 2020 [46]	National Cancer Institute’s Surveillance	R	2,903,241	First primary cancers	1877	0.064	63.1	1303 M, 574 F	To describe the risk of developing a second primary cancer among survivors of 10 cancer sites with the highest survival rates in the United States	1462 tongue, 343 floor, 72 salivary glands	3.8 years
Wu, 2020 [47]	People’s hospital of Nanjing, China	R	1161 (542 F, 619 M)	Pulmonary high-grade neuroendocrine carcinoma	13	1.12%	NR	NR	To determine the risk of second primary cancer in patients with pulmonary high-grade neuroendocrine carcinoma	floor of mouth, and gum and other mouth	16
				**Infectious Diseases**							
Song, 2019 [48]	The China Kadoorie Biobank	R	(a) 496,732 (203,660 M, 294,072 F) (b)37,336 (c) 97 (73 M, 24 F)	HBV	(a) 415 (b) no cases c) NR	(a) 1.98%/0.08% (b) no c) NR	(a) 51.5 (b)	NR	To assess the association between chronic HBV infection and risk of all cancer types	NR	(a) 8.85 (b)
Su, 2020 [49]	National Health insurance Research Database	P	100,058 (50,029 HCV-50,029 NO HCV) + 47,904 (23 952 therapy-23,952 no therapy)	HCV and anti-HCV therapy	229 (NO-HCV) 265 (HCV) + 146 (no therapy) 58 (therapy)	0.47%	59 (1 group)- 51 (2 group)	NR	To investigate the association between chronic hepatitis C and oral cancer, and the development of oral cancer after anti-hepatitis C virus (HCV) therapy	NR	7.9 years non-HCV/5.1 years HCV + 4.9 years no therapy/3.4 years therapy
Mahale, 2020 [50]	Surveillance, Epidemiology, and End Results (SEER)	R	531,460 (384,777 M, 146,683 F)	HIV+/lymphoid malignancies	511	0.01%	NR	NR	To describe the risk of cancers following lymphoid malignancies among HIV-infected people.	NR	NR
				**HSC**							
Yokota, 2010 [51]	Kanto Study Group for Cell Therapy	R	2062 (1225 M, 837 F)	Allogeneic hematopoietic SCT	10	35.7%/0.48%	42	5 M, 4 F, 1 Unknown	To evaluate the incidence and risk factors for secondary solid tumors in Japan after hematopoietic SCT	5 tongue, 3 gingiva, 2 oral mucosa	Median 5.7 years
Curtis, 2016 [52]	Center for International Blood and Marrow Transplant Research	P	24011	GVHD	24	13.11%/0.1%	NR	NR	To identify the number of patients with GVHD who developed second cancer	NR	30
Majhail, 2016 [53]	Center for International Blood and Marrow Transplant Research	R	4318 (2415 M, 1903 F)	Hematopoietic cell transplant	11	16.6%/0.25%	44	NR	To evaluate the risk of secondary solid cancers among allogeneic hematopoietic cell transplant recipients	NR	NR
Dyer, 2018 [54]	Blood and Marrow Transplant Network, Australia.	P	441 (191 F, 250 M)	Blood and marrow transplant	4	1.5%	NR	NR	To investigate oral health in blood and marrow transplant recipients	NR	12
Anak, 2018 [55]	Istanbul University Faculty of Istanbul Medicine, Our Children Leukemia Foundation BMT Center	P	24 (12 M, 12 F)	Hematopoietic cell transplantation in Fanconi Anemia patients	4	21	NR	To investigate SCC development after HSCT and examine features of the follow-up patients	4 retromolar trigone	NR	NR
Santarone, 2020 [56]	Bone marrow transplant center, Ospedale civile, Pescara, Italy	R	908 (498 M, 410 F)	Hematopoietic cell transplantation	12	100%/1.32%	47	8 M, 4 F	To demonstrate that oral cGVHD and a diagnosis of non-malignant hematologic disease are strong risk factors in the SOC development	6 tongue, 1 lower lip, 3 cheek mucosa, 1 gingival fornix, 1 hard palate	Unknown
				**Inflammatory** **Diseases**							
Bensing, 2008 [57]	National Death Register/Swedish Cancer Register	R	3299 (1359 M, 1940 F)	Autoimmune primary adrenocortical insufficiency	10	0.30%		NR	To assess the increased death risk and altered cancer incidence in patients with autoimmune primary adrenocortical insufficiency	NR	29 years
Zhang, 2009 [58]	Peking Union Medical College Hospital	R	1320 (1201 F, 119 M)	Sjögren’s syndrome	3	10%	50.7	NR	To identify the incidence of malignancy in primary Sjögren’s syndrome	2 tongue, 1 parotid gland	4.4 (mean)
Katsanos KH, 2015 [59]	Clinical Gastroenterology and Hepatology, NT.; USA	R	7294 (3785 F, 3509 M)	Inflammatory bowel disease (IBD)	11	0.15%	44.6	4 F, 7 M	To identify the number of patients with IBD that developed oral cancer	6 tongue, 2 hard palate, 3 buccal	NR
Rautemaa, 2016 [60]	Helsinki Hospital, Finland	R	92 (47 F, 45 M)	APECED	6	6.52%	37	2 F, 4 M	To study the possible association of APECED with oral and esophageal carcinoma.	buccal mucosa	NR
Derk, 2019 [21]	Thomas Jefferson University Philadelphia, Pennsylvania, USA	P	769	Systemic sclerosis	9	1.17%	49.2	NR	To describe the incidence of carcinoma of the tongue in a cohort of patients with systemic sclerosis	tongue	16
				**NR**							
Atsuta, 2014 [61]	Transplant Registry Unified Management Program	R	17545 (10,386 M, 7149 F)	NR	64	23.80%	NR	NR	To determine the incidence and the risk factors for secondary solid tumors after allogenic stem cell transplantation	NR	NR

NR: Not reported, P: Prospective, R: Retrospective.

**Table 2:** GRADE summary of findings for meta-analysis on immunosuppression and oral cancer incidence.

Quality Assessment, Outcome: Oral Cancer Incidence in Patients with Immunosuppression						
Question: Does the Immunosuppression Condition Have Influence on Oral Cancer Incidence?						
Number of Studies according to meta-analysis	Study design	Risk of Bias	Inconsistency	Indirectness	Imprecision	Publication bias
Meta-analysis on data from national registers (Figure 7): 23 studies	Cohort studies	Serious	Serious ^a^	Not Serious	Serious ^b^	Detected (1 study)
Meta-analysis on data not from national registers (Figure 8): 14 studies	Cohort studies	Serious	Not Serious	Not Serious	Serious ^b^	Undetected
Meta-analysis on GVHD patients (Figure 9): 5 studies	Cohort studies	Serious	Serious ^a^	Not Serious	Serious ^b^	Detected (1 study)

^a^. Due to high heterogeneity across studies. ^b^. Due to wide confidence intervals.

## Data Availability

Data are available upon request to the corresponding author.

## Supplementary Materials

The following supporting information can be downloaded at: https://www.mdpi.com/article/10.3390/cancers15123077/s1, Table S1. Full-text articles excluded, with reason [64,65,66,67,68,69,70,71,72,73,74,75,76,77,78,79,80,81,82,83,84,85,86,87,88,89,90,91,92,93,94,95,96,97,98,99,100,101,102,103,104,105,106,107,108,109,110,111,112,113,114,115,116,117,118,119,120,121,122,123,124,125,126,127,128,129,130,131,132,133,134,135,136,137,138,139,140,141,142,143,144,145,146,147,148,149,150,151,152,153,154,155,156,157,158,159,160,161,162,163,164,165,166,167,168,169,170,171,172,173,174,175,176,177,178,179,180,181,182,183,184,185,186,187,188,189,190,191,192,193,194,195,196,197,198,199,200,201,202,203,204,205,206,207,208,209,210,211,212,213,214,215,216,217,218].

## References

[1] Rivera C. (2015). Essentials of oral cancer. Int. J. Clin. Exp. Pathol..

[2] Liu J., Kaplon A., Blackman E., Miyamoto C., Savior D., Ragin C. (2020). The Impact of the Multidisciplinary Tumor Board on Head and Neck Cancer Outcomes. Laryngoscope.

[3] Shanti R.M., O’Malley B.W. (2018). Surgical Management of Oral Cancer. Dent. Clin. N. Am..

[4] Burnet M. (1964). Immunological factors in the process of carcinogenesis. Br. Med. Bull..

[5] Swann J.B., Smyth M.J. (2007). Immune surveillance of tumors. J. Clin. Investig..

[6] Bird L. (2016). Innate surveillance. Nat. Rev. Immunol..

[7] Lodish H. (2004). Molecular Biology of the Cell.

[8] Chinen J., Shearer W.T. (2010). Secondary immunodeficiencies, including HIV infection. J. Allergy Clin. Immunol..

[9] Notarangelo L.D., Bacchetta R., Casanova J.L., Su H.C. (2020). Human inborn errors of immunity: An expanding universe. Sci. Immunol..

[10] Rice J.M., Baan R.A., Stewart B.W., Straif K. (2019). Immunosuppression. Tumour Site Concordance and Mechanisms of Carcinogenesis.

[11] Geisser E.K. (2009). Immunosuppression. Cancer Treat. Res..

[12] Kumar M. (2016). Oral cancer: Etiology and risk factors: A review. J. Cancer Res. Ther..

[13] Vial T., Descotes J. (2003). Immunosuppressive drugs and cancer. Toxicology.

[14] Landis J.R., Koch G.G. (1977). The measurement of observer agreement for categorical data. Biometrics.

[15] Wells G.A. (2014). The Newcastle-Ottawa Scale (NOS) for Assessing the Quality of Nonrandomised studies in Meta-Analyses. Eur. J. Epidemiol..

[16] Guyatt G.H. (2008). GRADE: An emerging consensus on rating quality of evidence and strength of recommendations. BMJ.

[17] Higgins J.P.T. (2003). Measuring in consistency in meta-analyses. BMJ.

[18] Wallace B.C. (2012). Closing the Gap between Methodologists and End-Users: R as a Computational Back-End. J. Stat. Softw..

[19] Spolidorio L.C. (2006). Oral health in renal transplant recipients administered cyclosporin A or tacrolimus. Oral Dis..

[20] Jiang Y., Villeneuve P.J., Wielgosz A., Schaubel D.E., Fenton S.S., Mao Y. (2010). The incidence of cancer in a population-based cohort of Canadian heart transplant recipients. Am. J. Transpl..

[21] Derk C.T., Rasheed M., Spiegel J.R., Jimenez S.A. (2005). Increased incidence of carcinoma of the tongue in patients with systemic sclerosis. J. Rheumatol..

[22] Da Silva L.C., de Almeida Freitas R., de Andrade M.P., Piva M.R., Martins-Filho P.R., de Santana Santos T. (2012). Oral lesions in renal transplant. J. Craniofac. Surg..

[23] Öhman J., Rexius H., Mjörnstedt L., Gonzalez H., Holmberg E., Dellgren G., Hasséus B. (2015). Oral and lip cancer in solid organ transplant patients—A cohort study from a Swedish Transplant Centre. Oral. Oncol..

[24] Narayan G. (2018). Carcinoma of the Tongue in Renal Transplant Recipients: An Unusual Spectrum of De Novo Malignancy at a Tertiary Care Center in India over a Period of 26 Years. Indian J. Nephrol..

[25] Jäämaa-Holmberg S., Salmela B., Lemström K., Pukkala E., Lommi J. (2019). Cancer incidence and mortality after heart transplantation—A population-based national cohort study. Acta. Oncol..

[26] Laprise C., Cahoon E.K., Lynch C.F., Kahn A.R., Copeland G., Gonsalves L., Madeleine M.M., Pfeiffer R.M., Engels E.A. (2019). Risk of lip cancer after solid organ transplantation in the United States. Am. J. Transpl..

[27] Lin N.C., Chen Y.L., Tsai K.Y. (2019). Head and neck cancer in living donor liver transplant recipients: Single center retrospective study. Medicine.

[28] Johns M.E., Shikhani A.H., Kashima H.K., Matanoski G.M. (1986). Multiple primary neoplasms in patients with salivary gland or thyroid gland tumors. Laryngoscope.

[29] Gutman M. (1991). Are malignant melanoma patients at higher risk for a second cancer?. Cancer.

[30] Lishner M. (1991). Second malignant neoplasms in patients with non Hodgkin’s lymphoma. Hematol. Oncol..

[31] Hiyama T., Hanai A., Fujimoto I. (1991). Second primary cancer after diagnosis of stomach cancer in Osaka, Japan. Jpn. J. Cancer Res..

[32] Spitz M.R., Sider J.G., Schantz S.P., Newell G.R. (1992). Association between malignancies of the upper aerodigestive tract and uterine cervix. Head Neck.

[33] Rabkin C.S., Biggar R.J., Melbye M., Curtis R.E. (1992). Second primary cancers following anal and cervical carcinoma: Evidence of shared etiologic factors. Am. J. Epidemiol..

[34] Levi F., Randimbison L., La Vecchia C., Erler G., Te V.C. (1997). Incidence of invasive cancers following squamous cell skin cancer. Am. J. Epidemiol..

[35] Levi F., Randimbison L., Te V.C., La Vecchia C. (1999). Second primary cancers in patients with lung carcinoma. Cancer.

[36] Levi F., Randimbison L., Maspoli M., Te V.C., La Vecchia C. (2007). Second neoplasms after oesophageal cancer. Int. J. Cancer.

[37] Chuang S.C., Hashibe M., Scelo G., Brewster D.H., Pukkala E., Friis S., Tracey E., Weiderpass E., Hemminki K., Tamaro S. (2008). Risk of second primary cancer among esophageal cancer patients: A pooled analysis of 13 cancer registries. Cancer Epidemiol. Biomark. Prev..

[38] Brown A.P., Neeley E.S., Werner T., Soisson A.P., Burt R.W., Gaffney D.K. (2010). A population-based study of subsequent primary malignancies after endometrial cancer: Genetic, environmental, and treatment-related associations. Int. J. Radiat. Oncol. Biol. Phys..

[39] Zhu G., Chen Y., Zhu Z., Lu L., Bi X., Deng Q., Chen X., Su H., Liu Y., Guo H. (2012). Risk of second primary cancer after treatment for esophageal cancer: A pooled analysis of nine cancer registries. Dis. Esophagus.

[40] Hsu C.H., Huang C.L., Hsu Y.H., Iqbal U., Nguyen P.A., Jian W.S. (2014). Co-occurrence of second primary malignancy in patients with thyroid cancer. QJM.

[41] Robsahm T.E., Karagas M.R., Rees J.R., Syse A. (2014). New malignancies after squamous cell carcinoma and melanomas: A population-based study from Norway. BMC Cancer.

[42] Davis E.J., Beebe-Dimmer J.L., Yee C.L., Cooney K.A. (2014). Risk of second primary tumors in men diagnosed with prostate cancer: A population-based cohort study. Cancer.

[43] Bae S.H. (2016). Other primary systemic cancers in patients with melanoma: Analysis of balanced acral and nonacral melanomas. J. Am. Acad. Dermatol..

[44] Krilaviciute A., Vincerzevskiene I., Smailyte G. (2016). Basal cell skin cancer and the risk of second primary cancers: A cancer registry-based study in Lithuania. Ann. Epidemiol..

[45] Schlieve T., Heidel R.E., Carlson E.R. (2016). Second Primary Head and Neck Cancers after Non-Head and Neck Primary Cancers. J. Oral. Maxillofac. Surg..

[46] Adjei Boakye E., Wang M., Sharma A., Jenkins W.D., Osazuwa-Peters N., Chen B., Lee M., Schootman M. (2020). Risk of second primary cancers in individuals diagnosed with index smoking- and non-smoking- related cancers. J. Cancer Res. Clin. Oncol..

[47] Wu X., Zhang X., Tao L., Chen P. (2020). Risk of second primary malignancy in adults with pulmonary high-grade neuroendocrine carcinoma (HGNEC). BMC Cancer.

[48] Song C., Lv J., Liu Y., Chen J.G., Ge Z., Zhu J., Dai J., Du L.B., Yu C., Guo Y. (2019). Virus Infection and Risk of All Cancer Types. JAMA Netw. Open..

[49] Su T.H., Tseng T.C., Liu C.J., Chou S.W., Liu C.H., Yang H.C., Chen P.J., Chen D.S., Chen C.L., Kao J.H. (2020). Antiviral therapy against chronic hepatitis C is associated with a reduced risk of oral cancer. Int. J. Cancer.

[50] Mahale P., Ugoji C., Engels E.A., Shiels M.S., Peprah S., Morton L.M. (2020). Cancer risk following lymphoid malignancies among HIV-infected people. AIDS.

[51] Yokota A., Ozawa S., Masanori T., Akiyama H., Ohshima K., Kanda Y., Takahashi S., Mori T., Nakaseko C., Onoda M. (2012). Secondary solid tumors after allogeneic hematopoietic SCT in Japan. Bone Marrow Transpl..

[52] Curtis R.E., Metayer C., Rizzo J.D., Socié G., Sobocinski K.A., Flowers M.E., Travis W.D., Travis L.B., Horowitz M.M., Deeg H.J. (2005). Impact of chronic GVHD therapy on the development of squamous-cell cancers after hematopoietic stem-cell transplantation: An international case-control study. Blood.

[53] Majhail N.S., Brazauskas R., Rizzo J.D., Sobecks R.M., Wang Z., Horowitz M.M., Bolwell B., Wingard J.R., Socie G. (2011). Secondary solid cancers after allogeneic hematopoietic cell transplantation using busulfan-cyclophosphamide conditioning. Blood.

[54] Dyer G., Brice L., Schifter M., Gilroy N., Kabir M., Hertzberg M., Greenwood M., Larsen S.R., Moore J., Gottlieb D. (2018). Oral health and dental morbidity in long-term allogeneic blood and marrow transplant survivors in Australia. Aust. Dent. J..

[55] Anak S., Yalman N., Bilgen H., Sepet E., Deviren A., Gürtekin B., Tunca F., Başaran B. (2020). Squamous cell carcinoma development in Fanconi anemia patients who underwent hematopoietic stem cell transplantation. Pediatr. Transpl..

[56] Santarone S., Natale A., Angelini S., Papalinetti G., Vaddinelli D., Di Bartolomeo A., Di Bartolomeo P. (2021). Secondary oral cancer following hematopoietic cell transplantation. Bone Marrow Transpl..

[57] Bensing S., Brandt L., Tabaroj F., Sjöberg O., Nilsson B., Ekbom A., Blomqvist P., Kämpe O. (2008). Increased death risk and altered cancer incidence pattern in patients with isolated or combined autoimmune primary adrenocortical insufficiency. Clin. Endocrinol..

[58] Zhang W., Feng S., Yan S., Zhao Y., Li M., Sun J., Zhang F.C., Cui Q., Dong Y. (2010). Incidence of malignancy in primary Sjogren’s syndrome in a Chinese cohort. Rheumatology.

[59] Katsanos K.H., Roda G., McBride R.B., Cohen B., Colombel J.F. (2016). Increased Risk of Oral Cancer in Patients with Inflammatory Bowel Diseases. Clin. Gastroenterol. Hepatol..

[60] Rautemaa R., Hietanen J., Niissalo S., Pirinen S., Perheentupa J. (2007). Oral and oesophageal squamous cell carcinoma—A complication or component of autoimmune polyendocrinopathy-candidiasis-ectodermal dystrophy (APECED, APS-I). Oral Oncol..

[61] Atsuta Y., Suzuki R., Yamashita T., Fukuda T., Miyamura K., Taniguchi S., Iida H., Uchida T., Ikegame K., Takahashi S. (2014). Continuing increased risk of oral/esophageal cancer after allogeneic hematopoietic stem cell transplantation in adults in association with chronic graft-versus-host disease. Ann. Oncol..

[62] López-Pintor R.M., Hernández G., de Arriba L., de Andrés A. (2011). Lip cancer in renal transplant patients. Oral Oncol..

[63] Motlokwa P.K., Tsima B.M., Martei Y.M., Ralefala T., Galebole F., Stephens-Shields A.J., Grover S., Gross R. (2022). Disparities in Oral Cancer Stage at Presentation in a High HIV Prevalence Setting in Sub-Saharan Africa. JCO Glob. Oncol..

[64] Whiteside T.L. (2001). Immunobiology and immunotherapy of head and neck cancer. Curr Oncol Rep..

[65] Wen B.W., Tsai C.S., Lin C.L., Chang Y.J., Lee C.F., Hsu C.H., Kao C.H. (2014). Cancer risk among gingivitis and periodontitis patients: A nationwide cohort study. QJM.

[66] Gupta B., Ariyawardana A., Johnson N.W. (2013). Oral cancer in India continues in epidemic proportions: Evidence base and policy initiatives. Int. Dent. J..

[67] Dickenson A.J., Currie W.J., Avery B.S. (1995). Screening for syphilis in patients with carcinoma of the tongue. Br. J. Oral Maxillofac. Surg..

[68] Mohd Bakri M., Mohd Hussaini H., Rachel Holmes A., David Cannon R., Mary Rich A. (2010). Revisiting the association between candidal infection and carcinoma, particularly oral squamous cell carcinoma. J. Oral Microbiol..

[69] Garrote L.F., Herrero R., Reyes R.M., Vaccarella S., Anta J.L., Ferbeye L., Muñoz N., Franceschi S. (2001). Risk factors for cancer of the oral cavity and oro-pharynx in Cuba. Br. J. Cancer.

[70] Hassona Y., Scully C., Almangush A., Baqain Z., Sawair F. (2014). Oral potentially malignant disorders among dental patients: A pilot study in Jordan. Asian Pac. J. Cancer Prev..

[71] Ben-David Y., Leiser Y., Kachta O., El-Naaj I.A. (2013). Does long-term treatment with Doxil® predispose patients to oral cancer?. Int. J. Clin. Oncol..

[72] Fahmy M.S., Sadeghi A., Behmard S. (1983). Epidemiologic study of oral cancer in Fars Province, Iran. Community Dent. Oral Epidemiol..

[73] Sankaranarayanan R., Nair M.K., Mathew B., Balaram P., Sebastian P., Dutt S.C. (1992). Recent results of oral cancer research in Kerala, India. Head Neck..

[74] Warnakulasuriya S., Kovacevic T., Madden P., Coupland V.H., Sperandio M., Odell E., Møller H. (2011). Factors predicting malignant transformation in oral potentially malignant disorders among patients accrued over a 10-year period in South East England. J. Oral Pathol. Med..

[75] Túri K., Barabás P., Csurgay K., Léhner G.Y., Lőrincz A., Németh Z.S. (2013). An analysis of the epidemiological and etiological factors of oral tumors of young adults in a Central-Eastern European population. Pathol. Oncol. Res..

[76] Gorsky M., Epstein J.B., Oakley C., Le N.D., Hay J., Stevenson-Moore P. (2004). Carcinoma of the tongue: A case series analysis of clinical presentation, risk factors, staging, and outcome. Oral Surg. Oral Med. Oral Pathol. Oral Radiol. Endod..

[77] Hsue S.S., Wang W.C., Chen C.H., Lin C.C., Chen Y.K., Lin L.M. (2007). Malignant transformation in 1458 patients with potentially malignant oral mucosal disorders: A follow-up study based in a Taiwanese hospital. J. Oral Pathol. Med..

[78] Saira, Ahmed R., Malik S., Khan M.F., Khattak M.R. (2019). Epidemiological and clinical correlates of oral squamous cell carcinoma in patients from north-west Pakistan. J. Pak. Med. Assoc..

[79] Yao J.G., Gao L.B., Liu Y.G., Li J., Pang G.F. (2008). Genetic variation in interleukin-10 gene and risk of oral cancer. Clin. Chim. Acta.

[80] De Benedittis M., Petruzzi M., Giardina C., Lo Muzio L., Favia G., Serpico R. (2004). Oral squamous cell carcinoma during long-term treatment with hydroxyurea. Clin. Exp. Dermatol..

[81] Tsai C.W., Chang W.S., Lin K.C., Shih L.C., Tsai M.H., Hsiao C.L., Yang M.D., Lin C.C., Bau D.T. (2014). Significant association of Interleukin-10 genotypes and oral cancer susceptibility in Taiwan. Anticancer Res..

[82] Abhinav R.P., Williams J., Livingston P., Anjana R.M., Mohan V. (2020). Burden of diabetes and oral cancer in India. J. Diabetes Complicat..

[83] Satheeshkumar P.S., Mohan M.P. (2013). Oral Helicobacter pylori infection and the risk of oral cancer. Oral Oncol..

[84] Krüger M., Hansen T., Kasaj A., Moergel M. (2013). The Correlation between Chronic Periodontitis and Oral Cancer. Case Rep. Dent..

[85] Hermsen M.A., Xie Y., Rooimans M.A., Meijer G.A., Baak J.P., Plukker J.T., Arwert F., Joenje H. (2001). Cytogenetic characteristics of oral squamous cell carcinomas in Fanconi anemia. Fam. Cancer.

[86] Moura L.K.B., Mobin M., Matos F.T.C., Monte T.L., Lago E.C., Falcão C.A.M., Ferraz M.Â.A.L., Santos T.C., Tapety F.I., Nunes C.M.C.L.L. (2017). Bibliometric Analysis on the Risks of Oral Cancer for People Living with HIV/AIDS. Iran J. Public Health.

[87] Singh P.K., Ahmad M.K., Kumar V., Gupta R., Kohli M., Jain A., Mahdi A.A., Bogra J., Chandra G. (2017). Genetic polymorphism of interleukin-10 (-A592C) among oral cancer with squamous cell carcinoma. Arch. Oral Biol..

[88] Tarvainen L., Suojanen J., Kyyronen P., Lindqvist C., Martinsen J.I., Kjaerheim K., Lynge E., Sparen P., Tryggvadottir L., Weiderpass E. (2017). Occupational Risk for Oral Cancer in Nordic Countries. Anticancer Res..

[89] Soulier J. (2011). Fanconi anemia. Hematol. Am. Soc. Hematol. Educ. Program..

[90] Sun C., Hu Z., Zhong Z., Jiang Y., Sun R., Fei J., Xi Y., Li X., Song M., Li W. (2014). Clinical and prognostic analysis of second primary squamous cell carcinoma of the tongue after radiotherapy for nasopharyngeal carcinoma. Br. J. Oral Maxillofac. Surg..

[91] Hashibe M., Ritz B., Le A.D., Li G., Sankaranarayanan R., Zhang Z.F. (2005). Radiotherapy for oral cancer as a risk factor for second primary cancers. Cancer Lett..

[92] Santos A.M., Marcu L.G., Wong C.M., Bezak E. (2016). Risk estimation of second primary cancers after breast radiotherapy. Acta Oncol..

[93] Rafferty M.A., O’Dwyer T.P. (2001). Secondary primary malignancies in head and neck squamous cell carcinoma. J. Laryngol. Otol..

[94] Lee K.D., Lu C.H., Chen P.T., Chan C.H., Lin J.T., Huang C.E., Chen C.C., Chen M.C. (2009). The incidence and risk of developing a second primary esophageal cancer in patients with oral and pharyngeal carcinoma: A population-based study in Taiwan over a 25 year period. BMC Cancer.

[95] Shanmugham J.R., Zavras A.I., Rosner B.A., Giovannucci E.L. (2010). Alcohol-folate interactions in the risk of oral cancer in women: A prospective cohort study. Cancer Epidemiol. Biomark. Prev..

[96] De Araújo R.L., Lyko Kde F., Funke V.A., Torres-Pereira C.C. (2014). Oral cancer after prolonged immunosuppression for multiorgan chronic graft-versus-host disease. Rev. Bras. Hematol. Hemoter..

[97] Rosenquist K., Wennerberg J., Schildt E.B., Bladström A., Göran Hansson B., Andersson G. (2005). Oral status, oral infections and some lifestyle factors as risk factors for oral and oropharyngeal squamous cell carcinoma. A population-based case-control study in southern Sweden. Acta Otolaryngol..

[98] Douglas C.M., Jethwa A.R., Hasan W., Liu A., Gilbert R., Goldstein D., De Almedia J., Lipton J., Irish J.C. (2020). Long-term survival of head and neck squamous cell carcinoma after bone marrow transplant. Head Neck..

[99] Farrar M., Sandison A., Peston D., Gailani M. (2004). Immunocytochemical analysis of AE1/AE3, CK 14, Ki-67 and p53 expression in benign, premalignant and malignant oral tissue to establish putative markers for progression of oral carcinoma. Br. J. Biomed. Sci..

[100] Hsu H.J., Yang Y.H., Shieh T.Y., Chen C.H., Kao Y.H., Yang C.F., Ko E.C. (2015). TGF-β1 and IL-10 single nucleotide polymorphisms as risk factors for oral cancer in Taiwanese. Kaohsiung J. Med. Sci..

[101] Danylesko I., Shimoni A. (2018). Second Malignancies after Hematopoietic Stem Cell Transplantation. Curr. Treat. Options Oncol..

[102] Adhikari J., Sharma P., Bhatt V.R. (2015). Risk of secondary solid malignancies after allogeneic hematopoietic stem cell transplantation and preventive strategies. Future Oncol..

[103] Demarosi F., Lodi G., Carrassi A., Soligo D., Sardella A. (2005). Oral malignancies following HSCT: Graft versus host disease and other risk factors. Oral Oncol..

[104] Manavoğlu O., Orhan B., Evrensel T., Karabulut Y., Ozkocaman V., Ozyardimci C. (1996). Second primary cancer due to radiotherapy and chemotherapy. J. Environ. Pathol. Toxicol. Oncol..

[105] Takeuchi Y., Onizawa K., Wakatsuki T., Yamagata K., Hasegawa Y., Yoshida H. (2006). Tongue cancer after bone marrow transplantation. Oral Oncol..

[106] Tomihara K., Dehari H., Yamaguchi A., Abe M., Miyazaki A., Nakamori K., Hareyama M., Hiratsuka H. (2009). Squamous cell carcinoma of the buccal mucosa in a young adult with history of allogeneic bone marrow transplantation for childhood acute leukemia. Head Neck.

[107] Kawano K., Goto H., Takahashi Y., Kaku Y., Oobu K., Yanagisawa S. (2007). Secondary Squamous Cell Carcinoma of the Oral Cavity in Young Adults after Hematopoietic Stem Cell Transplantation for Leukemia: Report of Two Cases with Human Papillomavirus Infection. Oral Sci. Int..

[108] Inamoto Y., Shah N.N., Savani B.N., Shaw B.E., Abraham A.A., Ahmed I.A., Akpek G., Atsuta Y., Baker K.S., Basak G.W. (2015). Secondary solid cancer screening following hematopoietic cell transplantation. Bone Marrow Transplant..

[109] Hernández G., Arriba L., Jiménez C., Bagán J.V., Rivera B., Lucas M., Moreno E. (2003). Rapid progression from oral leukoplakia to carcinoma in an immunosuppressed liver transplant recipient. Oral Oncol..

[110] Torres-Pereira C.C., Stramandinoli-Zanicotti R.T., Amenábar J.M., Sassi L.M., Galbiatti Pedruzzi P.A., Piazzetta C.M., Bonfim C. (2014). Oral squamous cell carcinoma in two siblings with Fanconi anemia after allogeneic bone marrow transplantation. Spec. Care Dentist.

[111] Alotaiby F., Song F., Boyce B.J., Cao D., Zhao Y., Lai J. (2018). Unusual Papillary Squamous Cell Carcinoma of the Tip of Tongue Presenting in a Patient Status Post Heart Transplant. Anticancer Res..

[112] Shiboski C.H., Schmidt B.L., Jordan R.C. (2005). Tongue and tonsil carcinoma: Increasing trends in the U.S. population ages 20–44 years. Cancer.

[113] Weng X., Xing Y., Cheng B. (2017). Multiple and Recurrent Squamous Cell Carcinoma of the Oral Cavity After Graft-Versus-Host Disease. J. Oral Maxillofac. Surg..

[114] Sharma R.N. (1964). Oral Carcinoma: A Clinical Study Of 122 Cases. J. Indian Med. Assoc..

[115] Fu X., Chen S., Chen W., Yang Z., Song M., Li H., Zhang H., Yao F., Su X., Liu T. (2018). Clinical analysis of second primary gingival squamous cell carcinoma after radiotherapy. Oral Oncol..

[116] García-Martín J.M., Varela-Centelles P., González M., Seoane-Romero J.M., Seoane J., García-Pola M.J., Panta P. (2019). Epidemiology of oral cancer. Oral Cancer Detection.

[117] Tao Y., Sturgis E.M., Huang Z., Sun Y., Dahlstrom K.R., Wei Q., Li G. (2018). A TGF-β1 genetic variant at the miRNA187 binding site significantly modifies risk of HPV16-associated oropharyngeal cancer. Int. J. Cancer.

[118] Madrid C., Scully C. (2012). Oral cancer: Comprehending the condition, causes, controversies, control and consequences. 17. Osteonecrosis. Dent. Update.

[119] Dhanuthai K., Rojanawatsirivej S., Thosaporn W., Kintarak S., Subarnbhesaj A., Darling M., Kryshtalskyj E., Chiang C.P., Shin H.I., Choi S.Y. (2018). Oral cancer: A multicenter study. Med. Oral Patol. Oral Cir. Bucal..

[120] Geng F., Wang Q., Li C., Liu J., Zhang D., Zhang S., Pan Y. (2019). Identification of Potential Candidate Genes of Oral Cancer in Response to Chronic Infection With Porphyromonas gingivalis Using Bioinformatical Analyses. Front Oncol..

[121] Pisani L.P., Estadella D., Ribeiro D.A. (2017). The Role of Toll Like Receptors (TLRs) in Oral Carcinogenesis. Anticancer Res..

[122] Okubo M., Kioi M., Nakashima H., Sugiura K., Mitsudo K., Aoki I., Taniguchi H., Tohnai I. (2016). M2-polarized macrophages contribute to neovasculogenesis, leading to relapse of oral cancer following radiation. Sci Rep..

[123] Dewan K., Kelly R.D., Bardsley P. (2014). A national survey of consultants, specialists and specialist registrars in restorative dentistry for the assessment and treatment planning of oral cancer patients. Br. Dent. J..

[124] Mukhopadhyaya R., Rao R.S., Fakih A.R., Gangal S.G. (1986). Detection of circulating immune complexes in patients with squamous cell carcinoma of the oral cavity. J. Clin. Lab. Immunol..

[125] Adewole R.A. (2002). Alcohol, smoking and oral cancer. A 10-year retrospective study at Base Hospital, Yaba. West Afr. J. Med..

[126] Kashyap T., Pramanik K.K., Nath N., Mishra P., Singh A.K., Nagini S., Rana A., Mishra R. (2018). Crosstalk between Raf-MEK-ERK and PI3K-Akt-GSK3β signaling networks promotes chemoresistance, invasion/migration and stemness via expression of CD44 variants (v4 and v6) in oral cancer. Oral Oncol..

[127] Moore S., Johnson N., Pierce A., Wilson D. (1999). The epidemiology of lip cancer: A review of global incidence and aetiology. Oral Dis..

[128] Ueda N., Kamata N., Hayashi E., Yokoyama K., Hoteiya T., Nagayama M. (1999). Effects of an anti-angiogenic agent, TNP-470, on the growth of oral squamous cell carcinomas. Oral Oncol..

[129] Chen Y., Wang X., Fang J. (2019). Mesenchymal stem cells participate in oral mucosa carcinogenesis by regulating T cell proliferation. Clin. Immunol..

[130] Kikuchi K., Noguchi Y., de Rivera M.W. (2016). Detection of Epstein-Barr virus genome and latent infection gene expression in normal epithelia, epithelial dysplasia, and squamous cell carcinoma of the oral cavity. Tumour Biol..

[131] Lenouvel D., González-Moles M.Á., Talbaoui A. (2020). An update of knowledge on PD-L1 in head and neck cancers: Physiologic, prognostic and therapeutic perspectives. Oral Dis..

[132] Brown A.M., Lally E.T., Frankel A., Harwick R., Davis L.W., Rominger C.J. (1975). The association of the IGA levels of serum and whole saliva with the progression of oral cancer. Cancer.

[133] Johnson N.W. (2001). Az oralis carcinomák etiológiája és rizikófaktorai, különös tekintettel a dohányzásra és az alkoholfogyasztásra [Aetiology and risk factors for oral cancer, with special reference to tobacco and alcohol use]. Magy Onkol..

[134] Adams S., Lin J., Brown D., Shriver C.D., Zhu K. (2016). Ultraviolet Radiation Exposure and the Incidence of Oral, Pharyngeal and Cervical Cancer and Melanoma: An Analysis of the SEER Data. Anticancer Res..

[135] Kurokawa H., Tsuru S., Okada M., Nakamura T., Kajiyama M. (1993). Evaluation of tumor markers in patients with squamous cell carcinoma in the oral cavity. Int. J. Oral Maxillofac. Surg..

[136] Talamini R., Vaccarella S., Barbone F. (2000). Oral hygiene, dentition, sexual habits and risk of oral cancer. Br. J. Cancer.

[137] La Rosa G.R.M., Gattuso G., Pedullà E., Rapisarda E., Nicolosi D., Salmeri M. (2020). Association of oral dysbiosis with oral cancer development. Oncol. Lett..

[138] Das D., Ghosh S., Maitra A. (2019). Epigenomic dysregulation-mediated alterations of key biological pathways and tumor immune evasion are hallmarks of gingivo-buccal oral cancer. Clin. Epigenetics.

[139] Rajkumar T., Sridhar H., Balaram P. (2003). Oral cancer in Southern India: The influence of body size, diet, infections and sexual practices. Eur. J. Cancer Prev..

[140] Khanna S. (2008). Immunological and biochemical markers in oral carcinogenesis: The public health perspective. Int. J. Environ. Res. Public Health.

[141] Engku Nasrullah Satiman E.A.F., Ahmad H., Ramzi A.B., Wahab R.A., Kaderi M.A., Harun W.H.A.W., Dashper S. (2020). The role of Candida albicans candidalysin ECE1 gene in oral carcinogenesis. J. Oral Pathol. Med..

[142] Wu T.S., Tan C.T., Chang C.C. (2015). B-cell lymphoma/leukemia 10 promotes oral cancer progression through STAT1/ATF4/S100P signaling pathway. Oncogene..

[143] Malinowska K., Morawiec-Sztandera A., Majsterek I., Kaczmarczyk D. (2016). TC2 C776G polymorphism studies in patients with oral cancer in the Polish population. Pol. J. Pathol..

[144] Leuci S., Coppola N., Blasi A. (2020). Oral Dysplastic Complications after HSCT: Single Case Series of Multidisciplinary Evaluation of 80 Patients. Life.

[145] Anqi C., Takabatake K., Kawai H., Oo M.W., Yoshida S., Fujii M., Omori H., Sukegawa S., Nakano K., Tsuijigiwa H. (2019). Differentiation and roles of bone marrow-derived cells on the tumor microenvironment of oral squamous cell carcinoma. Oncol. Lett..

[146] Furquim C.P., Pivovar A., Amenábar J.M., Bonfim C., Torres-Pereira C.C. (2018). Oral cancer in Fanconi anemia: Review of 121 cases. Crit. Rev.Oncol. Hematol..

[147] Shah A.T., Wu E., Wein R.O. (2013). Oral squamous cell carcinoma in post-transplant patients. Am. J. Otolaryngol..

[148] Elad S., Zadik Y., Zeevi I., Miyazaki A., de Figueiredo M.A., Or R. (2010). Oral cancer in patients after hematopoietic stem-cell transplantation: Long-term follow-up suggests an increased risk for recurrence. Transplantation.

[149] Abdelsayed R.A., Sumner T., Allen C.M., Treadway A., Ness G.M., Penza S.L. (2002). Oral precancerous and malignant lesions associated with graft-versus-host disease: Report of 2 cases. Oral Surg. Oral Med. Oral Pathol. Oral Radiol. Endod..

[150] González-Moles M.Á., Ruiz-Ávila I., González-Ruiz L., Ayén Á., Gil-Montoya J.A., Ramos-García P. (2019). Malignant transformation risk of oral lichen planus: A systematic review and comprehensive meta-analysis. Oral Oncol..

[151] Nagao Y., Sata M., Fukuizumi K., Harada H., Kameyama T. (1996). Oral cancer and hepatitis C virus (HCV): Can HCV alone cause oral cancer?—A case report. Kurume Med. J..

[152] Nagao Y., Sata M., Noguchi S. (2000). Detection of hepatitis C virus RNA in oral lichen planus and oral cancer tissues. J. Oral Pathol. Med..

[153] Nagao Y., Sata M., Tanikawa K., Itoh K., Kameyama T. (1995). High prevalence of hepatitis C virus antibody and RNA in patients with oral cancer. J. Oral Pathol. Med..

[154] Nagao Y., Sata M. (2013). Oral verrucous carcinoma arising from lichen planus and esophageal squamous cell carcinoma in a patient with hepatitis C virus-related liver cirrhosis-hyperinsulinemia and malignant transformation: A case report. Biomed. Rep..

[155] Gandolfo S., Richiardi L., Carrozzo M. (2004). Risk of oral squamous cell carcinoma in 402 patients with oral lichen planus:A follow-up study in an Italian population. Oral Oncol..

[156] Wang L.Y., You S.L., Lu S.N. (2003). Risk of hepatocellular carcinoma and habits of alcohol drinking, betel quid chewing and cigarette smoking: A cohort of 2416 HBsAg-seropositive and 9421 HBsAg-seronegative male residents in Taiwan. Cancer Causes Control.

[157] Kao C.H., Sun L.M., Liang J.A., Chang S.N., Sung F.C., Muo C.H. (2012). Relationship of zolpidem and cancer risk: A Taiwanese population-based cohort study. Mayo Clin. Proc..

[158] Tandle A.T., Sanghvi V., Saranath D. (2001). Determination of p53 genotypes in oral cancer patients from India. Br. J. Cancer.

[159] Shih L.C., Li C.H., Sun K.T. (2018). Association of Matrix Metalloproteinase-7 Genotypes to the Risk of Oral Cancer in Taiwan. Anticancer Res..

[160] Chiu C.F., Tsai M.H., Tseng H.C., Wang C.L., Tsai F.J., Lin C.C., Bau D.T. (2008). A novel single nucleotide polymorphism in ERCC6 gene is associated with oral cancer susceptibility in Taiwanese patients. Oral Oncol..

[161] Park J.Y., Schantz S.P., Stern J.C., Kaur T., Lazarus P. (1999). Association between glutathione S-transferase pi genetic polymorphisms and oral cancer risk. Pharmacogenetics.

[162] Hatagima A., Costa E.C., Marques C.F., Koifman R.J., Boffetta P., Koifman S. (2008). Glutathione S-transferase polymorphisms and oral cancer: A case-control study in Rio de Janeiro, Brazil. Oral Oncol..

[163] Misra C., Majumder M., Bajaj S., Ghosh S., Roy B., Roychoudhury S. (2009). Polymorphisms at p53, p73, and MDM2 loci modulate the risk of tobacco associated leukoplakia and oral cancer. Mol Carcinog..

[164] Sartor M., Steingrimsdottir H., Elamin F. (1999). Role of p16/MTS1, cyclin D1 and RB in primary oral cancer and oral cancer cell lines. Br. J. Cancer.

[165] Chiang C.T., Chang T.K., Hwang Y.H. (2011). A critical exploration of blood and environmental chromium concentration among oral cancer patients in an oral cancer prevalent area of Taiwan. Environ. Geochem. Health.

[166] Chen W.C., Chen M.F., Lin P.Y. (2014). Significance of DNMT3b in oral cancer. PLoS ONE.

[167] Kang D., Gridley G., Huang W.Y. (2005). Microsatellite polymorphisms in the epidermal growth factor receptor (EGFR) gene and the transforming growth factor-alpha (TGFA) gene and risk of oral cancer in Puerto Rico. Pharmacogenet. Genom..

[168] Rao A.K.D.M., Manikandan M., Arunkumar G., Revathidevi S., Vinothkumar V., Arun K., Tiwary B.K., Rajkumar K.S. (2017). Prevalence of p53 codon 72, p73 G4C14-A4T14 and MDM2 T309G polymorphisms and its association with the risk of oral cancer in South Indians. Gene Rep..

[169] Yen C.Y., Liu S.Y., Chen C.H., Tseng H.F., Chuang L.Y., Yang C.H., Lin Y.C., Wen C.H., Chiang W.F., Ho C.H. (2008). Combinational polymorphisms of four DNA repair genes XRCC1, XRCC2, XRCC3, and XRCC4 and their association with oral cancer in Taiwan. J. Oral Pathol. Med..

[170] Ramachandran S., Ramadas K., Hariharan R., Rejnish Kumar R., Radhakrishna Pillai M. (2006). Single nucleotide polymorphisms of DNA repair genes XRCC1 and XPD and its molecular mapping in Indian oral cancer. Oral Oncol..

[171] Shukla D., Dinesh Kale A., Hallikerimath S., Vivekanandhan S., Venkatakanthaiah Y. (2012). Genetic polymorphism of drug metabolizing enzymes (GSTM1 and CYP1A1) as risk factors for oral premalignant lesions and oral cancer. Biomed. Pap. Med. Fac. Univ. Palacky Olomouc. Czech Repub..

[172] Park J.Y., Muscat J.E., Ren Q. (1997). CYP1A1 and GSTM1 polymorphisms and oral cancer risk. Cancer Epidemiol. Biomark. Prev..

[173] Cha I.H., Park J.Y., Chung W.Y., Choi M.A., Kim H.J., Park K.K. (2007). Polymorphisms of CYP1A1 and GSTM1 genes and susceptibility to oral cancer. Yonsei Med. J..

[174] Carneiro N.K., Oda J.M., Losi Guembarovski R., Ramos G., Oliveira B.V., Cavalli I.J., Ribeiro E.M.d.S.F., Goncalves M.S.B., Watanabe M.A.E. (2013). Possible association between TGF-β1 polymorphism and oral cancer. Int. J. Immunogenet..

[175] Wang L.-H., Ting S.-C., Chen C.-H., Tsai C.-C., Lung O., Liu T.-C., Lee C.-W., Wang Y.-Y., Tsai C.-L., Lin Y.-C. (2010). Polymorphisms in the apoptosis-associated genes FAS and FASL and risk of oral cancer and malignant potential of oral premalignant lesions in a Taiwanese population. J. Oral Pathol. Med..

[176] Chung T.T., Pan M.S., Kuo C.L. (2011). Impact of RECK gene polymorphisms and environmental factors on oral cancer susceptibility and clinicopathologic characteristics in Taiwan. Carcinogenesis.

[177] Shukla D., Dinesh Kale A., Hallikerimath S., Yerramalla V., Subbiah V., Mishra S. (2013). Association between GSTM1 and CYP1A1 polymorphisms and survival in oral cancer patients. Biomed. Pap. Med. Fac. Univ. Palacky Olomouc. Czech Repub..

[178] Gunduz E., Gunduz M., Ouchida M. (2005). Genetic and epigenetic alterations of BRG1 promote oral cancer development. Int. J. Oncol..

[179] Merrill R.M., Isakson R.T., Beck R.E. (2007). The association between allergies and cancer: What is currently known?. Ann. Allergy Asthma Immunol..

[180] Li G., Sturgis E.M., Wang L.E. (2004). Association of a p73 exon 2 G4C14-to-A4T14 polymorphism with risk of squamous cell carcinoma of the head and neck. Carcinogenesis.

[181] Twu C.W., Jiang R.S., Shu C.H., Lin J.C. (2006). Association of p53 codon 72 polymorphism with risk of hypopharyngeal squamous cell carcinoma in Taiwan. J. Formos. Med. Assoc..

[182] Katoh T. (1994). The frequency of glutathione-S-transferase M1 (GSTM1) gene deletion in patients with lung and oral cancer. Sangyo Igaku..

[183] Wang Z. (2005). The role of COX-2 in oral cancer development, and chemoprevention/ treatment of oral cancer by selective COX-2 inhibitors. Curr. Pharm. Des..

[184] Liu F., Liu L., Li B. (2011). p73 G4C14-A4T14 polymorphism and cancer risk: A meta-analysis based on 27 case-control studies. Mutagenesis.

[185] Singh A.P., Shah P.P., Ruwali M., Mathur N., Pant M.C., Parmar D. (2009). Polymorphism in cytochrome P4501A1 is significantly associated with head and neck cancer risk. Cancer Investig..

[186] Shillitoe E.J. (2009). The role of viruses in squamous cell carcinoma of the oropharyngeal mucosa. Oral Oncol..

[187] Beppu M., Ikebe T., Shirasuna K. (2002). The inhibitory effects of immunosuppressive factors, dexamethasone and interleukin-4, on NF-kappaB-mediated protease production by oral cancer. Biochim. Biophys. Acta.

[188] Van der Meij E.H., Epstein J.B., Hay J., Ho V., Lerner K. (1996). Sweet’s syndrome in a patient with oral cancer associated with radiotherapy. Eur. J. Cancer B Oral Oncol..

[189] Uittamo J., Siikala E., Kaihovaara P., Salaspuro M., Rautemaa R. (2009). Chronic candidosis and oral cancer in APECED-patients: Production of carcinogenic acetaldehyde from glucose and ethanol by Candida albicans. Int. J. Cancer.

[190] Shillitoe E.J. (1976). The role of immunology in the diagnosis, prognosis and treatment planning of oral cancer. Proc. R. Soc. Med..

[191] Meurman J.H. (2010). Infectious and dietary risk factors of oral cancer. Oral Oncol..

[192] Sanjaya P.R., Gokul S., Gururaj Patil B., Raju R. (2011). Candida in oral pre-cancer and oral cancer. Med. Hypotheses.

[193] Morris L.G., Patel S.G., Shah J.P., Ganly I. (2010). Squamous cell carcinoma of the oral tongue in the pediatric age group: A matched-pair analysis of survival. Arch. Otolaryngol. Head Neck Surg..

[194] Hara H., Ozeki S., Nagata T., Okamoto M., Sasaguri M., Tashiro H., Jingu K. (1988). Pulmonary tuberculosis in patients with oral cancer. Gan No Rinsho..

[195] Laprise C., Shahul H.P., Madathil S.A. (2016). Periodontal diseases and risk of oral cancer in Southern India: Results from the HeNCe Life study. Int. J. Cancer..

[196] Arantes D.A., Costa N.L., Mendonça E.F., Silva T.A., Batista A.C. (2016). Overexpression of immunosuppressive cytokines is associated with poorer clinical stage of oral squamous cell carcinoma. Arch. Oral Biol..

[197] Hwang P.H., Lian L., Zavras A.I. (2012). Alcohol intake and folate antagonism via CYP2E1 and ALDH1: Effects on oral carcinogenesis. Med. Hypotheses.

[198] Mun M., Yap T., Alnuaimi A.D., Adams G.G., McCullough M.J. (2016). Oral candidal carriage in asymptomatic patients. Aust. Dent. J..

[199] Krogh P., Hald B., Holmstrup P. (1987). Possible mycological etiology of oral mucosal cancer: Catalytic potential of infecting Candida albicans and other yeasts in production of N-nitrosobenzylmethylamine. Carcinogenesis.

[200] Yakin M., Gavidi R.O., Cox B., Rich A. (2017). Oral cancer risk factors in New Zealand. N. Z. Med. J..

[201] Sheu J.J., Keller J.J., Lin H.C. (2012). Increased risk of cancer after Bell’s palsy: A 5-year follow-up study. J. Neurooncol..

[202] Ma’aita J.K. (2000). Oral cancer in Jordan: A retrospective study of 118 patients. Croat. Med. J..

[203] Mäkinen A., Nawaz A., Mäkitie A., Meurman J.H. (2018). Role of Non-Albicans Candida and Candida Albicans in Oral Squamous Cell Cancer Patients. J. Oral Maxillofac. Surg..

[204] Menicagli R., Bolla G., Menicagli L., Esseridou A. (2017). The Possible Role of Diabetes in the Etiology of Laryngeal Cancer. Gulf. J. Oncolog..

[205] Bhattathiri N.V., Bindu L., Remani P., Chandralekha B., Nair K.M. (1998). Radiation-induced acute immediate nuclear abnormalities in oral cancer cells: Serial cytologic evaluation. Acta Cytol..

[206] D’Costa J., Saranath D., Sanghvi V., Mehta A.R. (1998). Epstein-Barr virus in tobacco-induced oral cancers and oral lesions in patients from India. J. Oral Pathol. Med..

[207] Jin X., Lu S., Xing X., Wang L., Mu D., He M., Huang H., Zeng X., Chen Q. (2013). Thalidomide: Features and potential significance in oral precancerous conditions and oral cancer. J. Oral Pathol. Med..

[208] Gall F., Colella G., Di Onofrio V., Rossiello R., Angelillo I.F., Liguori G. (2013). Candida spp. in oral cancer and oral precancerous lesions. New Microbiol..

[209] Vijayakumar T., Sasidharan V.K., Ankathil R., Remani P., Kumari T.V., Vasudevan D.M. (1984). Incidence of hepatitis B surface antigen (HBsAg) in oral cancer and carcinoma of uterine cervix. Indian J. Cancer.

[210] Li M.H., Ito D., Sanada M., Odani T., Hatori M., Iwase M., Nagumo M. (2004). Effect of 5-fluorouracil on G1 phase cell cycle regulation in oral cancer cell lines. Oral Oncol..

[211] Mawardi H., Elad S., Correa M.E., Stevenson K., Woo S.B., Almazrooa S., Haddad R., Antin H.J., Soiffer R., Treister H. (2011). Oral epithelial dysplasia and squamous cell carcinoma following allogeneic hematopoietic stem cell transplantation: Clinical presentation and treatment outcomes. Bone Marrow Transplant..

[212] Gruter M.O., Brand H.S. (2020). Oral health complications after a heart transplant: A review. Br. Dent. J..

[213] Sankaranarayanan R., Dinshaw K., Nene B.M. (2006). Cervical and oral cancer screening in India. J. Med. Screen..

[214] Nagasaka M., Zaki M., Kim H. (2016). PD1/PD-L1 inhibition as a potential radiosensitizer in head and neck squamous cell carcinoma: A case report. J. Immunother. Cancer.

[215] Kapoor V., Aggarwal S., Das S.N. (2016). 6-Gingerol Mediates its Anti- Tumor Activities in Human Oral and Cervical Cancer Cell Lines through Apoptosis and Cell Cycle Arrest. Phytother. Res..

[216] Ha N.H., Park D.G., Woo B.H. (2016). Porphyromonas gingivalis increases the invasiveness of oral cancer cells by upregulating IL-8 and MMPs. Cytokine.

[217] Ahmed H.G. (2013). Aetiology of oral cancer in the Sudan. J. Oral Maxillofac. Res..

[218] Lucchese A. (2015). Viruses and Oral Cancer: Crossreactivity as a Potential Link. Anticancer Agents Med. Chem..

