# Supplementary material for: Is Systemic Immunosuppression a Risk Factor for Oral Cancer? A Systematic Review and Meta-Analysis
Source: Cancers (Basel). 2023 Jun 6;15(12):3077. doi: 10.3390/cancers15123077 (PMC10296244; doi:10.3390/cancers15123077)
Supplement: Supplementary file 1 [file cancers-15-03077-s001.zip › cancers-2400660-supplementary.pdf]

Table S1. Full-text articles excluded, with reason [64–218].

STUDY	Reason for exclusion
Immunobiology and immunotherapy of head and neck cancer.<br>Whiteside T L, 2001	Review
Immunohistochemical analysis of dendritic cell in oral squamous cell carcinoma.<br>Wang Zhi-yong, 2004	Not Included Language
Cyclooxygenase-2 (COX-2) expression in high-risk premalignant oral lesions.<br>Subdø John, 2003	Retracted Article
Unusual malignant tumours in 49 patients with HIV infection.<br>Monfardini S, 1989	No Pertinent Study
Solid tumors associated with HIV infection.<br>Crosato I M, 1993	No Pertinent Study
Systemic lupus erythematosus is a risk factor for cancer: a nationwide population-based study in Korea<br>Bae E H, 2019	No Pertinent Study
Association of non-acquired immunodeficiency syndrome-defining cancers with human immunodeficiency virus infection.<br>Rabkin C S, 1998	Review
Serum antioxidant vitamins and the risk of oral cancer in patients seen at a tertiary institution in Nigeria.<br>Lawal A O, 2012	No Pertinent Study
Multiple and Recurrent Squamous Cell Carcinoma of the Oral Cavity After Graft-Versus-Host Disease.<br>Weng Xiuhong, 2017	Less Than 10 Patients
Oral conditions associated with hepatitis C virus infection.<br>Alavian Seyed-Moayed, 2013	No Pertinent Study
Malignant tumors following renal transplantation.<br>Végso Gyula, 2007	Not Included Language
Nutrition: impact on oral and systemic health.<br>Enwonwu C O, 2001	No Pertinent Study
Recurrence of oral cancer in patients with radio-osteomyelitis of the jaws.<br>Nwoku A L, 1973	No Pertinent Study
Alcohol-folate interactions in the risk of oral cancer in women: a prospective cohort study.<br>Shanmugham Jayapriyaa, 2010	No Pertinent Study
Prevalence of cervical, oral, and anal human papillomavirus infection in women living with HIV in Denmark - The SHADE cohort study.<br>Kristina Thorsteinsson, 2018	No Pertinent Study
Mitochondrial DNA copy number and risk of oral cancer: a report from Northeast India.<br>Mondal Rosy, 2013	No Pertinent Study
Bionutrition and oral cancer in humans.<br>Enwonwu, 1995	No Pertinent Study
Diabetes and oral oncogenesis.	No Pertinent Study

Vairaktaris Eleftherios, 2007	
Impact of dietary vitamin D on initiation and progression of oral cancer.<br>Aparajita Verma, 2020	No Pertinent Study
Role of Oral Microbial Infections in Oral Cancer.<br>Ferguson Brett, 2017	No Pertinent Study
The Oral Microbiome in Health and Its Implication in Oral and Systemic Diseases.<br>Sampaio-Maia, 2016	No Pertinent Study
High incidence of multiple primary carcinomas in HCV-infected patients with oral squamous cell carcinoma.<br>Nagao Yumiko, 2009	No Pertinent Study
Folate intake and risk of oral and pharyngeal cancer.<br>Pelucchi C, 2003	No Pertinent Study
An inverse association of Helicobacter pylori infection with oral squamous cell carcinoma.<br>Meng Xuw, 2003	No Pertinent Study
Diabetes and oral tumors in Hungary: epidemiological correlations.<br>Ujpàl Marta, 2004	Not Clinical Study
No evidence of association of xenotropic murine leukemia virus-related virus with oral cancers: Experience from a tertiary care center in South India.<br>Sinha M, 2015	No Pertinent Study
Risk factors for oropharynx cancer in a cohort of HIV-infected veterans.<br>Chew Erin, 2017<br>.	No Pertinent Study
Possible mycological etiology of oral mucosal cancer: catalytic potential of infecting Candida albicans and other yeasts in production of N-nitrosobenzylmethylamine.<br>Krogh, 1987	No Pertinent Study
Increased risk of cancer after Bell's palsy: a 5-year follow-up study.<br>Jau-Jiuan Sheu, 2017	No Pertinent Study
The Possible Role of Diabetes in the Etiology of Laryngeal Cancer.<br>Menicagli R, 2017	No Pertinent Study
Cancer risk among gingivitis and periodontitis patients: a nationwide cohort study.<br>Ben W, 2014	No Pertinent Study
Oral cancer in India continues in epidemic proportions: evidence base and policy initiatives.<br>Gupta Bhawna, 2013	No Pertinent Study
Screening for syphilis in patients with carcinoma of the tongue.<br>Dickenson AJ, 1995	No Pertinent Study
Revisiting the association between candidal infection and carcinoma, particularly oral squamous cell carcinoma.<br>Bakri Marina, 2010	Not Clinical Study
Risk factors for cancer of the oral cavity and oro-pharynx in Cuba.<br>Garrote L F, 2001	Not Clinical Study
Oral potentially malignant disorders among dental patients: a pilot study in Jordan.<br>Hassona Y, 2014	No Pertinent Study
Does long-term treatment with Doxil¬Æ predispose patients to oral cancer?	No Pertinent Study

Yehuda Ben David, 2013	
Epidemiologic study of oral cancer in Fars Province, Iran<br>Fahmi M S, 1983	Not Clinical Study
Recent results of oral cancer research in Kerala, India.<br>R Sankaranarayanan, 1992	Not Clinical Study
Factors predicting malignant transformation in oral potentially malignant disorders among patients accrued over a 10-year period in South East England.<br>S Warnakulasuriya, 2011	No Pertinent Study
An analysis of the epidemiological and etiological factors of oral tumors of young adults in a Central-Eastern European population.<br>Túri K, 2013	Not Clinical Study
Carcinoma of the tongue: a case series analysis of clinical presentation, risk factors, staging, and outcome<br>Gorski Meir, 2004<br>.	Not Clinical Study
Malignant transformation in 1458 patients with potentially malignant oral mucosal disorders: a follow-up study based in a Taiwanese hospital.<br>Shue-Sang Hsue, 2007.	No Pertinent Study
Epidemiological and clinical correlates of oral squamous cell carcinoma in patients from north-west Pakistan.<br>Saira et al., 2019	Not Clinical Study
Genetic variation in interleukin-10 gene and risk of oral cancer.<br>Yao Jin-Guang et al., 2008	No Pertinent Study
Oral squamous cell carcinoma during long-term treatment with hydroxyurea.<br>De Benedettis M et al., 2004	No Pertinent Study
Significant association of Interleukin-10 genotypes and oral cancer susceptibility in Taiwan.<br>Tsai Chia-Wen et al., 2004	No Pertinent Study
Burden of diabetes and oral cancer in India.<br>Rajendra Prabhu Abhinav et al., 2020	No Pertinent Study
Oral Helicobacter pylori infection and the risk of oral cancer.<br>Satheeshkumar P S, 2013	No Pertinent Study
The Correlation between Chronic Periodontitis and Oral Cancer.<br>Krüger M et al., 2013	No Pertinent Study
Cytogenetic characteristics of oral squamous cell carcinomas in Fanconi anemia.<br>Hermsen M A et al, 2001	No Pertinent Study
Bibliometric Analysis on the Risks of Oral Cancer for People Living with HIV/AIDS.<br>Batista Moura et al., 2017	No Pertinent Study
Genetic polymorphism of interleukin-10 (-A592C) among oral cancer with squamous cell carcinoma.<br>Singh P K, 2017	No Pertinent Study
Occupational Risk for Oral Cancer in Nordic Countries.<br>Tarvainen L et al., 2017	No Pertinent Study
Oral potentially malignant disorders in a large dental population.<br>Villa A et al., 2014<br>Villa A, Gohel A. Oral potentially malignant disorders in a large dental population. J Appl Oral Sci.	No Pertinent Study

2014 Nov-Dec;22(6):473-6.	
Fanconi Anemia.<br>Soulier Jean, 2011	Review
Clinical and prognostic analysis of second primary squamous cell carcinoma of the tongue after radio-therapy for nasopharyngeal carcinoma.<br>Sun C, 2014	No Pertinent Study
Radiotherapy for oral cancer as a risk factor for second primary cancers.<br>Hashibe S et al., 2005	No Pertinent Study
Risk estimation of second primary cancers after breast radiotherapy.<br>Santons A M C et al., 2016	No Pertinent Study
Secondary primary malignancies in head and neck squamous cell carcinoma.<br>Rafferty A M et al., 2001	No Pertinent Study
The incidence and risk of developing a second primary esophageal cancer in patients withoral and pharyngeal carcinoma: a population-based study in Taiwan over a 25 year period.<br>Lee Kuan-Der et al., 2009	No Pertinent Study
Alcohol-Folate Interactions in the Risk of Oral Cancer in Women: A Prospective Cohort Study.<br>Jayapriyaa R Shanmugham et al., 2010	No Pertinent Study
Oral cancer after prolonged immunosuppression for multiorgan chronic graft-versus-host disease.<br>Renata Lins Fuentes de Araújo et al., 2014	Case Report
Oral status, oral infections and some lifestyle factors as risk factors for oral and oropharyngeal squa-mous cell carcinoma: a population-based case-control study in southern Sweden.<br>Rosenquist K et al., 2005	No Pertinent Study
Long-term survival of head and neck squamous cell carcinoma after bone marrow transplant.<br>Douglas C M et. Al, 2020	No Pertinent Study
Immunocytochemical analysis of AE1/AE3, CK 14; Ki-67 and p53 expression in benign, premalignant and malignant oral tissue to establish putative markers for progression of oral carcinoma.<br>Farrar M et. Al, 2004	No Pertinent Study
TGF- β1 and IL-10 single nucleotide polymorphisms as risk factors for oral cancer in Taiwanese.<br>Hsu H J et al., 2005	No Pertinent Study
Second Malignancies after Hematopoietic Stem Cell Transplantation.<br>Danylesko I et al., 2018	Review
Risk of secondary solid malignancies after allogenic hematopoietic stem cell transplantation and pre-ventive strategies.<br>Adikhari J, 2015	Review
Oral malignancies following HSCT: Graft versus host disease and other risk factors.<br>Demarosi F et al., 2005	Review
Second primary cancer due to radiotherapy and chemotherapy.<br>Mavanoglu et al., 1996	Less Than 10 Patients
Tongue cancer after bone marrow transplantion.<br>Takeuchi Y et al., 2006	Less Than 10 Patients
Squamous cell carcinoma of the buccal mucosa in a young adult history of allogenic bone marrow transplantation for childhood acute leukemia.<br>Tomihara K et al., 2009	Case Report

Secondary squamous cell carcinoma of the oral cavity in young adults after hematopoietic stem cell transplantation for leukemia.<br>Kawano K et al., 2007	Less Than 10 Patients
Secondary solid cancer screening following hematopoietic cell transplantation.<br>Inamoto Y et al., 2015	Review
Rapid progression from oral leukoplakia to carcinoma in an immunosuppressed liver transplant recipient.<br>Hernandéz G et al., 2003	Case Report
Oral squamous cell carcinoma in two siblings with fanconi anemia after allogeneic bone marrow transplantation.<br>Cassius Carvalho Torres-Pereira et al., 2014	Less Than 10 Patients
Oral cancer after prolonged immunosuppression for multiorgan chronic Graft-versus-host disease.<br>Renata Lins Fuentes de Araújo et al., 2014	Case Report
Unusual papillary squamous cell carcinoma of the tip of tongue presenting in a patient status post heart transplant.<br>Alotaiby F et al., 2018	Case Report
Tongue and tonsil carcinoma: increasing trends in the U.S. population ages 20-44 years.<br>Shiboski C H et al., 2005	Review
Oral helicobacter pylori infection and the risk of oral cancer.<br>P S Satheeshkumar et al, 2013	No Pertinent Study
Does long-term treatment with doxil predispose patients to oral cancer?<br>Ben-David Y et al., 2013	No Pertinent Study
Multiple and reccurent squamous cell carcinoma of the oral cavity after Graft-versus-host disease.<br>Weng X et al., 2017	Less Than 10 Patients
ORAL CARCINOMA: A CLINICAL STUDY OF 122 CASES<br>Sharma R N, 1964	Not Pertinent Study
Clinical analysis of second primary gingival squamous cell carcinoma after radiotherapy.<br>Fu X et al., 2018	Case Report
Epidemiology of oral cancer.<br>García- Martin et al., 2019	Not Clinical Study
A TGF-Œ≤1 genetic variant at the miRNA187 binding site significantly modifies risk of HPV16-associated oropharyngeal cancer.<br>Tao ye et al., 2018	Not Pertinent Study
Oral cancer: comprehending the condition, causes, controversies, control and consequences. 17. Osteonecrosis.<br>Madrid C., 2012	Review
Oral cancer: A multicenter study<br>Dhanuthai K et al., 2018	Not Pertinent Study
Identification of Potential Candidate Genes of Oral Cancer in Response to Chronic Infection With Porphyromonas gingivalis Using Bioinformatical Analyses.<br>Geng F et al., 2019	Not Pertinent Study
The Role of Toll Like Receptors (TLRs) in Oral Carcinogenesis.<br>Pellegrini Pisani L et al., 2017	Not Pertinent Study

M2-polarized macrophages contribute to neovasculogenesis, leading to relapse of oral cancer following radiation<br>Okubo M et al., 2016	Not Pertinent Study
A national survey of consultants, specialists and specialist registrars in restorative dentistry for the assessment and treatment planning of oral cancer patients.<br>Dewan K et al., 2014	Not Pertinent Study
Detection of circulating immune complexes in patients with squamous cell carcinoma of the oral cavity.<br>Mukhopadhyaya R, 1986	Not Pertinent Study
Alcohol, smoking and oral cancer. A 10-year retrospective study at Base Hospital, Yaba<br>Adewole, 2002	Not Pertinent Study
Crosstalk between Raf-MEK-ERK and PI3K-Akt-GSK3Œ≤ signaling networks promotes chemo-resistance, invasion/migration and stemness via expression of CD44 variants (v4 and v6) in oral cancer.<br>Kashyap, 2018	Not Pertinent Study
Increased oral cancer risk after renal transplantation<br>R.M. Lopez-Pintor, et al	Grey Literature
The epidemiology of lip cancer: a review of global incidence and aetiology<br>Moore et al 1999	Review
Effects of an anti-angiogenic agent, TNP-470, on the growth of oral squamous cell carcinomas.<br>Ueda et al 1999	No Pertinent Study
Mesenchymal stem cells participate in oral mucosa carcinogenesis by regulating T cell proliferation.<br>Chen et al 2019	No Pertinent Study
Detection of Epstein-Barr virus genome and latent infection gene expression in normal epithelia, epithelial dysplasia, and squamous cell carcinoma of the oral cavity<br>Kikuchi et al 2016	No Pertinent Study
An update of knowledge on PD-L1 in head and neck cancers: Physiologic, prognostic and therapeutic perspectives<br>Lenouvel et al 2020	Review
The association of the IGA levels of serum and whole saliva with the progression of oral cancer.<br>Brown et al 1975	No Pertinent Study
Az oralis carcinomák etiológiája és rizikófaktorai, különös tekintettel a dohányzásra és az alkoholfogyasztásra<br>Johnson et al 2001	Not Included Language
Ultraviolet Radiation Exposure and the Incidence of Oral, Pharyngeal and Cervical Cancer and Melanoma: An Analysis of the SEER Data<br>Adams et al 2016	No Pertinent Study
Evaluation of tumor markers in patients with squamous cell carcinoma in the oral cavity<br>Kurokawa et al 1993	No Pertinent Study
Oral hygiene, dentition, sexual habits and risk of oral cancer<br>Talamini et al 2000	No Pertinent Study
Tsantoulis et al 2007<br>Tsantoulis PK, Kastrinakis NG, Tourvas AD, Laskaris G, Gorgoulis VG. Advances in the biology of oral cancer. *Oral Oncol*. 2007;43(6):523-534.	Review

Association of oral dysbiosis with oral cancer development.<br>La Rosa et al 2020	Review
Epigenomic dysregulation-mediated alterations of key biological pathways and tumor immune evasion are hallmarks of gingivo-buccal oral cancer<br>Das et al 2019	No Pertinent Study
Oral cancer in Southern India: the influence of body size, diet, infections and sexual practices<br>Rajkumar et al 2003	No Pertinent Study
Immunological and biochemical markers in oral carcinogenesis: the public health perspective<br>Khanna et al 2008	No Pertinent Study
The role of Candida albicans candidalysin ECE1 gene in oral carcinogenesis<br>Engku Nasrullah Satiman et al 2020	Review
B-cell lymphoma/leukemia 10 promotes oral cancer progression through STAT1/ATF4/S100P signal-ing pathway<br>Wu et al 2015	No Pertinent Study
TC2 C776G polymorphism studies in patients with oral cancer in the Polish population<br>Malinowska et al 2016	Less Than 10 Patients
Oral Dysplastic Complications after HSCT: Single Case Series of Multidisciplinary Evaluation of 80 Patients.<br>Leuci et al 2020	No Pertinent Study
Differentiation and roles of bone marrow-derived cells on the tumor microenvironment of oral squa-mous cell carcinoma.<br>Anqi et al 2019	No Pertinent Study
Unusual Papillary Squamous Cell Carcinoma of the Tip of Tongue Presenting in a Patient Status Post Heart Transplant<br>Alotaiby et al 2018	Less Than 10 Patients
Oral cancer in Fanconi anemia: Review of 121 cases<br>Furguim et al 2018	Review
Multiple and Recurrent Squamous Cell Carcinoma of the Oral Cavity After Graft-Versus-Host Dis-ease.<br>Weng et al 2017	Less Than 10 Patients
Oral squamous cell carcinoma in post-transplant patients<br>Shah et al 2013	Review
Oral cancer in patients after hematopoietic stem-cell transplantation: long-term follow-up suggests an increased risk for recurrence<br>Elad et al 2010	Review
Oral precancerous and malignant lesions associated with graft-versus-host disease: report of 2 cases.<br>Abdelsayed et al 2002	Less Than 10 Patients
Malignant transformation risk of oral lichen planus: A systematic review and comprehensive meta-analysis.<br>González-Moles et al 2019	Meta-Analysis
Oral cancer and hepatitis C virus (HCV): can HCV alone cause oral cancer?--a case report.<br>Nagao et al 1996	Less Than 10 Patients
Detection of hepatitis C virus RNA in oral lichen planus and oral cancer tissues<br>Nagao et al 2000	No Pertinent Study

High prevalence of hepatitis C virus antibody and RNA in patients with oral cancer<br>Nagao et al 1995	No Pertinent Study
Oral verrucous carcinoma arising from lichen planus and esophageal squamous cell carcinoma in a patient with hepatitis C virus-related liver cirrhosis-hyperinsulinemia and malignant transformation: A case report<br>Nagao et al 2013	Less Than 10 Patients
Risk of oral squamous cell carcinoma in 402 patients with oral lichen planus: a follow-up study in an Italian population<br>Gandolfo et al 2004	No Pertinent Study
Risk of hepatocellular carcinoma and habits of alcohol drinking, betel quid chewing and cigarette smoking: a cohort of 2416 HBsAg-seropositive and 9421 HBsAg-seronegative male residents in Taiwan<br>Wang et al 2003	No Pertinent Study
Relationship of zolpidem and cancer risk: a Taiwanese population-based cohort study<br>Kao et al 2012	No Pertinent Study
Genetic variation in interleukin-10 gene and risk of oral cancer<br>Yao et al 2008	No Pertinent Study
Determination of p53 genotypes in oral cancer patients from India<br>Tandle et al 2001	No Pertinent Study
Association of Matrix Metalloproteinase-7 Genotypes to the Risk of Oral Cancer in Taiwan.<br>Shih et al 2018	No Pertinent Study
A novel single nucleotide polymorphism in ERCC6 gene is associated with oral cancer susceptibility in Taiwanese patients.<br>Chiu et al 2008	No Pertinent Study
Association between glutathione S-transferase pi genetic polymorphisms and oral cancer risk<br>Park et al 2000<br>.	No Pertinent Study
Glutathione S-transferase polymorphisms and oral cancer: a case-control study in Rio de Janeiro, Braziel<br>Hatagima et al 2008	No Pertinent Study
Polymorphisms at p53, p73, and MDM2 loci modulate the risk of tobacco associated leukoplakia and oral cancer<br>Misra et al 2009	No Pertinent Study
Role of p16/MTS1, cyclin D1 and RB in primary oral cancer and oral cancer cell lines<br>Sartor et al 1999	No Pertinent Study
A critical exploration of blood and environmental chromium concentration among oral cancer patients in an oral cancer prevalent area of Taiwan<br>Chiang et al 2011	No Pertinent Study
Significance of DNMT3b in oral cancer<br>Chen et al 2014	In Vitro Study
Microsatellite polymorphisms in the epidermal growth factor receptor (EGFR) gene and the transforming growth factor-alpha (TGFA) gene and risk of oral cancer in Puerto Rico<br>Kang et al 2005	No Pertinent Study
Prevalence of p53 codon 72, p73 G4C14-A4T14 and MDM2 T309G polymorphisms and its associa-	No Pertinent Study

tion with the risk of oral cancer in South Indians<br>Arunagiri et al 2017	
Combinational polymorphisms of four DNA repair genes XRCC1, XRCC2, XRCC3, and XRCC4 and their association with oral cancer in Taiwan<br>Yen et al 2008	In Vitro Study
Single nucleotide polymorphisms of DNA repair genes XRCC1 and XPD and its molecular mapping in Indian oral cancer<br>Ramachandran et al 2006	No Pertinent Study
Genetic polymorphism of drug metabolizing enzymes (GSTM1 and CYP1A1) as risk factors for oral premalignant lesions and oral cancer.<br>Shukla et al 2012	No Pertinent Study
CYP1A1 and GSTM1 polymorphisms and oral cancer risk<br>Park et al 1997	No Pertinent Study
Polymorphisms of CYP1A1 and GSTM1 genes and susceptibility to oral cancer<br>Cha et al 2007	No Pertinent Study
Possible association between TGF-β1 polymorphism and oral cancer<br>Carneiro et al 2013	No Pertinent Study
Polymorphisms in the apoptosis-associated genes FAS and FASL and risk of oral cancer and malignant potential of oral premalignant lesions in a Taiwanese population<br>Wang et al 2010	No Pertinent Study
Impact of RECK gene polymorphisms and environmental factors on oral cancer susceptibility and clinicopathologic characteristics in Taiwan<br>Chung et al 2011	No Pertinent Study
Association between GSTM1 and CYP1A1 polymorphisms and survival in oral cancer patients<br>Shukla et al 2013	No Pertinent Study
Genetic and epigenetic alterations of BRG1 promote oral cancer development<br>Gunduz et al 2005	No Pertinent Study
The association between allergies and cancer: what is currently known?.<br>Merrill et al 2007	Review
Association of a p73 exon 2 G4C14-to-A4T14 polymorphism with risk of squamous cell carcinoma of the head and neck<br>Li et al 2004	No Pertinent Study
Association of p53 codon 72 polymorphism with risk of hypopharyngeal squamous cell carcinoma in Taiwan<br>Twu et al 2006	No Pertinent Study
[The frequency of glutathione-S-transferase M1 (GSTM1) gene deletion in patients with lung and oral cancer]<br>Katoh et al 1994	Not Included Language
The role of COX-2 in oral cancer development, and chemoprevention/ treatment of oral cancer by selective COX-2 inhibitors<br>Wang et al 2005	Review
p73 G4C14-A4T14 polymorphism and cancer risk: a meta-analysis based on 27 case-control studies<br>Liu et al 2011	Meta-Analysis
Polymorphism in cytochrome P4501A1 is significantly associated with head and neck cancer risk	No Pertinent Study

Singh et al 2009	
The role of viruses in squamous cell carcinoma of the oropharyngeal mucosa<br>Shillitoe et al 2009	Review
The inhibitory effects of immunosuppressive factors, dexamethasone and interleukin-4, on NF-kappaB-mediated protease production by oral cancer<br>Beppu et al 2002	No Pertinent Study
Sweet's syndrome in a patient with oral cancer associated with radiotherapy.<br>Van der Meji et al 1996	Less Than 10 Patients
Chronic candidosis and oral cancer in APECED-patients: production of carcinogenic acetaldehyde from glucose and ethanol by Candida albicans<br>Uittamo et al 2009	No Pertinent Study
Squamous cell carcinoma of the buccal mucosa in a young adult with history of allogeneic bone marrow transplantation for childhood acute leukemia<br>Tomihara et al 2009	Less Than 10 Patients
The role of immunology in the diagnosis, prognosis and treatment planning of oral cancer.<br>Shillitoe et al 1976	Grey Literature
Infectious and dietary risk factors of oral cancer<br>Meurman et al 2010	Review
Candida in oral pre-cancer and oral cancer<br>Sanjaya et al 2011	No Pertinent Study
Squamous cell carcinoma of the oral tongue in the pediatric age group: a matched-pair analysis of survival<br>Morris et al 2010	Not Clinical Study
[Pulmonary tuberculosis in patients with oral cancer]<br>Hara et al 1988	Not Included Language
Periodontal diseases and risk of oral cancer in Southern India: Results from the HeNCe Life study<br>Laprise et al 2016	Not Included Language
Overexpression of immunosuppressive cytokines is associated with poorer clinical stage of oral squamous cell carcinoma<br>Arantes et al 2016	Not Clinical Study
Alcohol intake and folate antagonism via CYP2E1 and ALDH1: effects on oral carcinogenesis<br>Hwang et al 2012	No Pertinent Study
. Oral candidal carriage in asymptomatic patients.<br>Mun et al 2016	No Pertinent Study
Possible mycological etiology of oral mucosal cancer: catalytic potential of infecting Candida albicans and other yeasts in production of N-nitrosobenzylmethylamine.<br>Krogh et al 1987	No Pertinent Study
Oral cancer risk factors in New Zealand<br>Yakin et al 2017	No Clinical Study
Increased risk of cancer after Bell's palsy: a 5-year follow-up study<br>Sheu et al 2012	No Pertinent Study
Oral cancer in Jordan: a retrospective study of 118 patients<br>Ma'aita et al 2000	No Pertinent Study

Role of Non-Albicans Candida and Candida Albicans in Oral Squamous Cell Cancer Patients<br>Mäkinen et al 2018	No Pertinent Study
The Possible Role of Diabetes in the Etiology of Laryngeal Cancer<br>Menicagli et al 2017	No Pertinent Study
Revisiting the association between candidal infection and carcinoma, particularly oral squamous cell carcinoma<br>Mohd Bakri et al 2010	No Clinical Study
Radiation-induced acute immediate nuclear abnormalities in oral cancer cells: serial cytologic evaluation<br>Bhattathiri et al 1998	No Pertinent Study
Epstein-Barr virus in tobacco-induced oral cancers and oral lesions in patients from India<br>D'Costa et al 1998	No Pertinent Study
Thalidomide: features and potential significance in oral precancerous conditions and oral cancer.<br>Jin et al 2013	Review
Candida spp. in oral cancer and oral precancerous lesions.<br>Gall et al 2013	No Pertinent Study
Incidence of hepatitis B surface antigen (HBsAg) in oral cancer and carcinoma of uterine cervix<br>Vijayakumar et al 1984	No Pertinent Study
Effect of 5-fluorouracil on G1 phase cell cycle regulation in oral cancer cell lines<br>Li et al 2004	No Pertinent Study
Oral epithelial dysplasia and squamous cell carcinoma following allogeneic hematopoietic stem cell transplantation: clinical presentation and treatment outcomes<br>Mawardi et al 2011	Lack Of Data
Oral health complications after a heart transplant: a review<br>Gruter et al 2020	Review
Cervical and oral cancer screening in India<br>Sankaranarayanan et al 2006	No Pertinent Study
PD1/PD-L1 inhibition as a potential radiosensitizer in head and neck squamous cell carcinoma: a case report.<br>Nagasaka et al 2016	Less Than 10 Patients
6-Gingerol Mediates its Anti- Tumor Activities in Human Oral and Cervical Cancer Cell Lines through Apoptosis and Cell Cycle Arrest<br>Kapoor et al 2016	No Pertinent Study
Porphyromonas gingivalis increases the invasiveness of oral cancer cells by upregulating IL-8 and MMPs<br>Ha et al 2016	No Pertinent Study
Aetiology of oral cancer in the Sudan<br>Ahmed et al 2013	No Pertinent Study
Viruses and Oral Cancer: Crossreactivity as a Potential Link<br>Lucchese et al 2015	No Pertinent Study
